# Biochemical and structural characterization of a GNAT superfamily protein acetyltransferase from *Helicobacter pylori*

**DOI:** 10.1016/j.jbc.2025.110356

**Published:** 2025-06-10

**Authors:** Venkatareddy Dadireddy, Amrendra Kumar, Sumith Kumar, Siddhartha P. Sarma, Pranjal Mahanta, Suryanarayanarao Ramakumar, Rao N. Desirazu

**Affiliations:** 1 Department of Physics, Indian Institute of Science, Bangalore, India; 2 Department of Biochemistry, Indian Institute of Science, Bangalore, India; 3 Molecular Biophysics Unit, Indian Institute of Science, Bangalore, India; 4 School of Biological Sciences, National Institute of Science Education and Research, Bhubaneswar, India

**Keywords:** *Helicobacter pylori*, acetylation, protein acetyltransferase, post-translational modification, natural transformation, 3D structure, loop dynamics, HP0935, catalytic mechanism

## Abstract

*Helicobacter pylori* (*H. pylori*), a gastric pathogen with high genetic variability and a unique niche, causes peptic ulcers and gastric cancer. Natural transformation contributes to the genetic variability of *H. pylori*. To date, protein acetylation and the associated acetyltransferase(s) have not been reported in this bacterium. Here, we report protein acetylation in *H. pylori* and identify a putative protein acetyltransferase, HP0935, capable of acetylating amino acids and proteins, including DNA processing protein A (DprA), which is involved in natural transformation. HP0935 acetylates residue K133 in DprA, which is important for DNA binding, thus is likely to regulate natural transformation. We determined the crystal structures of HP0935 in its apo form and in complex with acetyl-coenzyme A (ACO) to 2.00 Å and 2.40 Å resolution, respectively. Structural analysis revealed a conformational change in the substrate-binding loops, **α**1-**α**2 and **β**6-**β**7, upon ACO binding. The structural comparison showed that HP0935 differs from other protein acetyltransferases in the length and orientation of these loops. Molecular dynamics simulation data suggest that these loops are highly dynamic, and ACO binding could affect their dynamics. Given that several proteins may undergo acetylation in *H. pylori* and the fact that HP0935 is the only known protein acetyltransferase, the loop dynamics are likely to facilitate the acceptance of multiple substrates by HP0935. Structure-based mutational analysis showed that no general base is required for the enzymatic activity. However, a conserved catalytic water molecule at the active site is likely to serve the purpose. Furthermore, the general acid Y127 is essential for enzymatic activity.

Protein acetylation is an essential post-translational modification (PTM) that is conserved in all three domains of life. It has several roles in key cellular processes including, gene regulation (1), metabolic pathways (2, 3), protein stability (4), protein-protein interactions (5), enzymatic activity (6), and virulence (7). Essentially, two amino groups, namely the N-terminal (Nα) and lysine side chain (Nε) of proteins, undergo acetylation. Nα-acetylation is irreversible and is catalyzed by N-terminal acetyltransferases (NATs). Conversely, Nε-acetylation is reversible, and the acetylated and unacetylated states are maintained by lysine acetyltransferases (KATs) and deacetylases, respectively. KATs are also called protein acetyltransferases (PATs), especially in bacteria. Protein acetyltransferases have been grouped into three families based on sequence similarity, domain organization, and substrate specificity—GNAT (Gcn5-related N-acetyltransferase), MYST (human MOZ, yeast Ybf2/Sas3, yeast Sas2, and human Tip60), and p300/CBP (human p300 and human CREB-binding protein) (8).

These three families of N-acetyltransferases employ different mechanisms for acetylation. However, the core of the N-acetyltransferases, which binds ACO through its pyrophosphate and pantetheine moieties, remains conserved. ACO's adenine base and acetyl moieties have no significant contribution to its binding to the N-acetyltransferase (9). These properties enable N-acetyltransferases to bind various acyl-coenzyme A as well as coenzyme A (COA) within their structure. Eukaryotes possess all three families, whereas prokaryotes are known to have only GNAT superfamily members. However, GNAT substrates range from small molecules to proteins (10). In bacteria, the number of GNATs encoded by a given genome varies, from one in *Borrelia burgdorferi* to approximately 72 in *Streptomyces coelicolor* (11). Although GNAT superfamily members exhibit low sequence similarity, the structure of the GNAT fold remains conserved. Based on the number and organization of domains, prokaryotic GNATs are classified into five types: I–V. Types I to III comprise a regulatory domain alongside the catalytic GNAT domain, type IV comprises only the catalytic domain, and type V comprises an N-terminal and central GNAT domain followed by a C-terminal region. However, it remains unclear whether the central GNAT domain in type V is catalytically active (11).

*Helicobacter pylori* (*H. pylori*) is a Gram-negative, microaerophilic, and spiral-shaped bacterium residing in the stomach. *H. pylori* is the causative agent of peptic ulcers, chronic gastritis, and gastric cancers (12, 13). Over half of the world’s population is infected with *H. pylori* (14), with up to 90% of infected individuals developing no symptoms of infection (15). *H. pylori* exhibits significant genetic diversity among strains due to insertions, deletions, point mutations, and phase variation (16).

This genetic diversity enables *H. pylori* strains to survive in the acidic environment of the human stomach (17). Genetic recombination can occur between different strains within an infected host (18). Natural competence and an efficient recombination system are the key contributors to horizontal gene transfer-mediated genetic diversity in *H. pylori*. The genetic diversity in the virulence genes and the host context determine the clinical outcome of *H. pylori* infection (16).

Until recently, PTMs have not been reported in *H. pylori*. A major virulence factor, CagA, undergoes phosphorylation by host kinase upon secretion into the host gastric epithelium (19). A proteomic study identified the α-subunit of urease with N-terminal acetyl modification (20). A recent study reported how the acetylation of RNase J affects its oligomeric state and consequently its activity (21). Given the ubiquitous nature of protein acetylation, we initially assessed the prevalence of protein acetylation in *H. pylori* and subsequently identified the relevant protein acetyltransferase, HP0935. A biochemical study showed that HP0935, the only known protein acetyltransferase in *H. pylori*, can acetylate free lysine amino acid and lysine residues in proteins. Based on the three-dimensional (3D) structure and dynamics, we hypothesize that the flexibility in substrate-binding loops enables HP0935 to accommodate and acetylate multiple protein substrates.

## Results and discussion

### Acetylation in *H. pylori*

The high genetic diversity among *H. pylori* strains may lead to differential expression of proteins and their PTMs. To investigate protein acetylation, whole cell lysates of different *H. pylori* strains—26695, J99, I-10, San-64, and 93(1)—were analyzed by Western blotting using an anti-acetyl lysine antibody (see Experimental procedures). Protein acetylation in *H. pylori* was prominent and varied significantly among the strains studied (Fig. S1). In strain 26695, protein acetylation seems well distributed from lower to higher-molecular-weight proteins. Higher-molecular-weight proteins were predominantly acetylated in strains I-10, San-64, and 93(1). However, in strain J99, lower-molecular-weight proteins were primarily acetylated. These observations suggest that the acetylome could depend on the genetic composition of a given strain. Several acetylome studies in other bacteria indicate its role in regulating various biological processes (22, 23, 24, 25). Therefore, protein acetylation in *H. pylori* suggests potential regulatory roles in *H. pylori* physiology.

### Identification of a putative N-acetyltransferase HP0935 from *H. pylori*

In prokaryotes, protein acetylation follows two distinct mechanisms: enzymatic acetylation catalyzed by protein acetyltransferases and non-enzymatic acetylation mediated by the highly unstable acetyl phosphate (AcP) and ACO. To identify putative protein acetyltransferase(s), a PSI-BLAST was carried out with the GNAT domain of known protein acetyltransferases from the organisms *Salmonella enterica*, *Saccharomyces cerevisiae*, *Mycobacterium smegmatis*, and *Mycobacterium tuberculosis* (see Experimental procedures). The search identified HP0935, an N-acetyltransferase domain-containing protein, from *H. pylori* strain 26695 with a significant expect (E)-value (Table S1). HP0935 shares the highest sequence identity of 27% (over 71 amino acids overlap) with protein acetyltransferases MSMEG_5458 from *M. smegmatis*. In addition to PSI-BLAST, InterProScan (26) identified two GNAT domain-containing proteins (InterPro ID: IPR000182), in strain 26695. These include UDP-4-amino-4,6-dideoxy-N-acetyl-β-L-altrosamine N-acetyltransferase (PseH, a pseudaminic acid biosynthesis protein) and N-acetyltransferase domain-containing protein, HP0935. Given that PseH is a small-molecule acetyltransferase involved in the biosynthesis of pseudaminic acid that is subsequently used to modify flagellin (27), we hypothesized that HP0935 is a probable protein acetyltransferase. HP0935 is a small (161 amino acids long) single GNAT domain-containing protein belonging to type IV protein acetyltransferases (11). For biochemical and structural characterization, the *hp0935* gene was cloned, and the protein was expressed and purified to homogeneity (see Supporting information, Fig. S2*A*). The protein’s identity was confirmed by peptide mass fingerprinting (Fig. S2*B*).

### Plausible substrate(s) of HP0935

**Figure 1:**
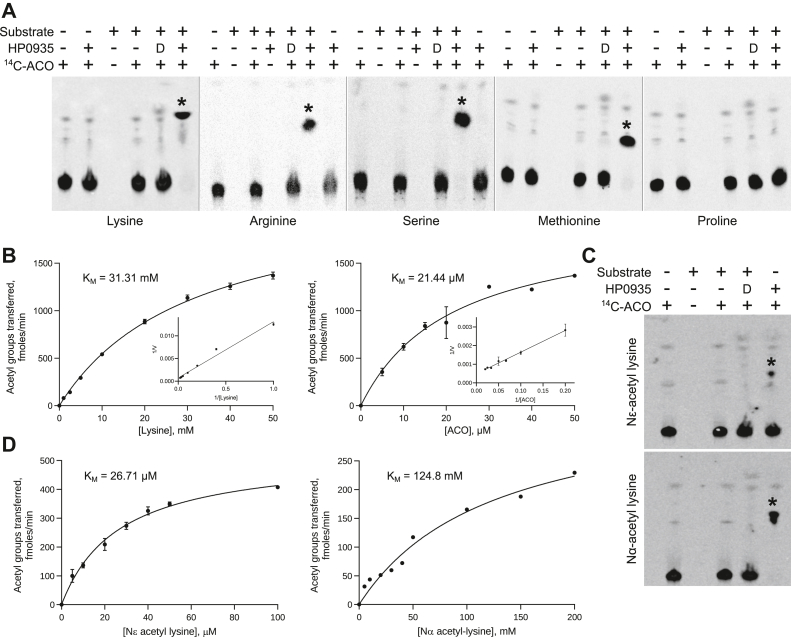
**Acetyltransferase activity of HP0935.***A*, acetyltransferase activity of HP0935 was probed using lysine, arginine, serine, methionine, and proline as substrates. The acetylation reaction mix was separated using silica gel thin-layer chromatography and detected by autoradiography. ^14^C-ACO (^14^C-labeled acetyl-coenzyme A) was used as an acetyl group donor. Acetylation products are indicated with an *asterisk* (∗). D: heat-denatured HP0935. *B*, HP0935 showed Michaelis-Menten kinetics. The kinetic parameters (K_M_ and V_max_) for lysine and ACO were measured at 800 nM HP0935 concentration. Reaction rates were quantified from Figure S3. *C*, the group-specific (Nα or Nϵ) acetylation in lysine was tested using Nα-acetyl lysine (free ϵ-amino group) and Nϵ-acetyl lysine (free α-amino group). The acetylation reaction mix was separated using silica gel thin-layer chromatography and detected by autoradiography. ^14^C-ACO is used as an acetyl group donor. Acetylation products are indicated with an *asterisk* (∗). *D*, heat-denatured HP0935. *D*, Michaelis-Menten kinetics of HP0935 (800 nM) for substrates Nϵ-acetyl lysine and Nα-acetyl lysine. The *error bars* represent the SD.

To investigate the acetyltransferase activity of HP0935, thin-layer chromatography (TLC) and autoradiography-based acetylation assays were performed with amino acids lysine, arginine, serine, methionine, and proline as substrates (see Experimental procedures). HP0935 exhibited activity towards lysine, arginine, serine, and methionine but not proline (Fig. 1*A*). Unlike *Salinococcus halodurans* lysine Nα-acetyltransferase, which acetylates only basic amino acids (28), HP0935 acetylates both neutral (serine and methionine) and basic amino acids, except proline. To determine the kinetic parameters, initial velocity measurements were conducted using TLC followed by densitometric analysis (see Experimental procedures, Fig. S3). HP0935 exhibited Michaelis-Menten (MM) kinetics, with apparent MM constant (K_M_) values of 31.31 mM for lysine and 21.44 μM for ACO (Fig. 1*B*). A similar result, a high K_M_ value for lysine compared to ACO, was observed for *S. cerevisiae* L-lysine N6-acetyltransferase (29).

To determine which of the primary amino groups, Nα or Nε, of lysine undergoes acetylation, a similar TLC-based assays were performed using Nε-acetyl lysine (free Nα group) and Nα-acetyl lysine (free Nε group) as substrates. HP0935 acetylated both the substrates, with a greater degree of acetylation observed for Nε-acetyl lysine, suggesting a preference for the Nα-amino group (Fig. 1*C*). Further assays with Nα-acetyl lysine and Nε-acetyl lysine also showed MM kinetics, revealing a significantly higher K_M_ for Nα-acetyl lysine (124.8 mM) compared to Nε-acetyl lysine (26.71 μM) (Fig. 1*D*). HP0935’s preference for the Nα-amino group was further confirmed by 1D-proton nuclear magnetic resonance (NMR) spectroscopy. Enzymatically acetylated lysine showed a chemical shift corresponding to that of Nα-acetyl lysine (Fig. S4), indicating HP0935 prefers the Nα-amino group over Nε. Our results show that HP0935 acetylates both Nα and Nε positions of free lysine but preferentially the former, suggesting that HP0935 could function as N-terminal and Nε-lysine protein acetyltransferase. A recent study reported a new family of plastid-localized GNATs with dual-specificity, *i.e.*, N-terminal and Nε-lysine acetylation of proteins (30). These dual-specificity acetyltransferases reveal a new layer of complexity in the machinery controlling protein acetylation.

D-amino acids are essential components of the bacterial peptidoglycan cell wall (31). Interestingly, HP0935 was found to be stereospecific, as D-lysine was not acetylated (Fig. S5*A*). Notably, the *S. cerevisiae* D-amino acid N-acetyltransferase, encoded by HPA3 (a putative histone/protein acetyltransferase), is a unique enzyme that acts specifically on D-amino acids but does little on histones (32).

A PSI-BLAST search revealed spermidine-N(1)-acetyltransferase from *Escherichia coli*, *Vibrio cholerae,* and *Salmonella typhi* shares ∼30% sequence identity (∼60% query cover) with HP0935. However, HP0935 exhibited no enzymatic acetylation of spermidine, showing only minimal non-enzymatic activity, thereby ruling out its role as a spermidine-N(1)-acetyltransferase (Fig. S5*B*). There are reports of GNAT protein acetyltransferases acetylating the Nε-amino group of lysine in proteins and small molecules. Eis, a protein acetyltransferase from *M. tuberculosis* (*Mtb*), was found to acetylate aminoglycoside antibiotics, contributing to drug resistance, and also aryl alkylamines like histamine and octopamine (33, 34). The known protein substrates of Eis include a human dual specificity protein phosphatase 16 (DUSP16)/mitogen-activated protein kinase phosphatase-7 (MKP-7) (33), and a nucleoid-associated protein HU of *Mtb* (35). In another study, Hpa3, a histone acetyltransferase, was shown to acetylate D-amino acids as well as histone H4 (36). Based on these observations, HP0935 is likely a protein acetyltransferase with the ability to acetylate free amino acids.

### HP0935 acetylates DprA

Further, acetylation of the Nα-amino group in proteins, *i.e.*, N-terminal acetylation, was investigated using peptides as substrates. Among the known N-terminal acetyltransferases in prokaryotes, RimI, RimJ, and RimL acetylate ribosomal proteins S18, S5, and L7/L12, respectively. Acetylation of EF-Tu (elongation factor-Tu), ribosomal protein S7, and glutamate dehydrogenase by unknown acetyltransferases in various organisms has also been reported (37, 38). Several prokaryotic N-terminal acetyltransferases preferentially acetylate methionine (M), serine (S), alanine (A), or threonine (T) at the protein N-terminus (37). We, therefore, wanted to investigate the Nα-acetylation activity of HP0935 with peptides containing the first amino acid as M, S, or A. No HP0935 activity was observed with any of these peptides (data not shown). However, a detailed investigation of HP0935 for its N-terminal acetyltransferase activity is needed. It is possible that, apart from the first N-terminal amino acid, the structure of a protein substrate might be necessary for HP0935 activity. In parallel, the Nε-acetylation activity of HP0935 was also investigated using protein substrates.

**Figure 2:**
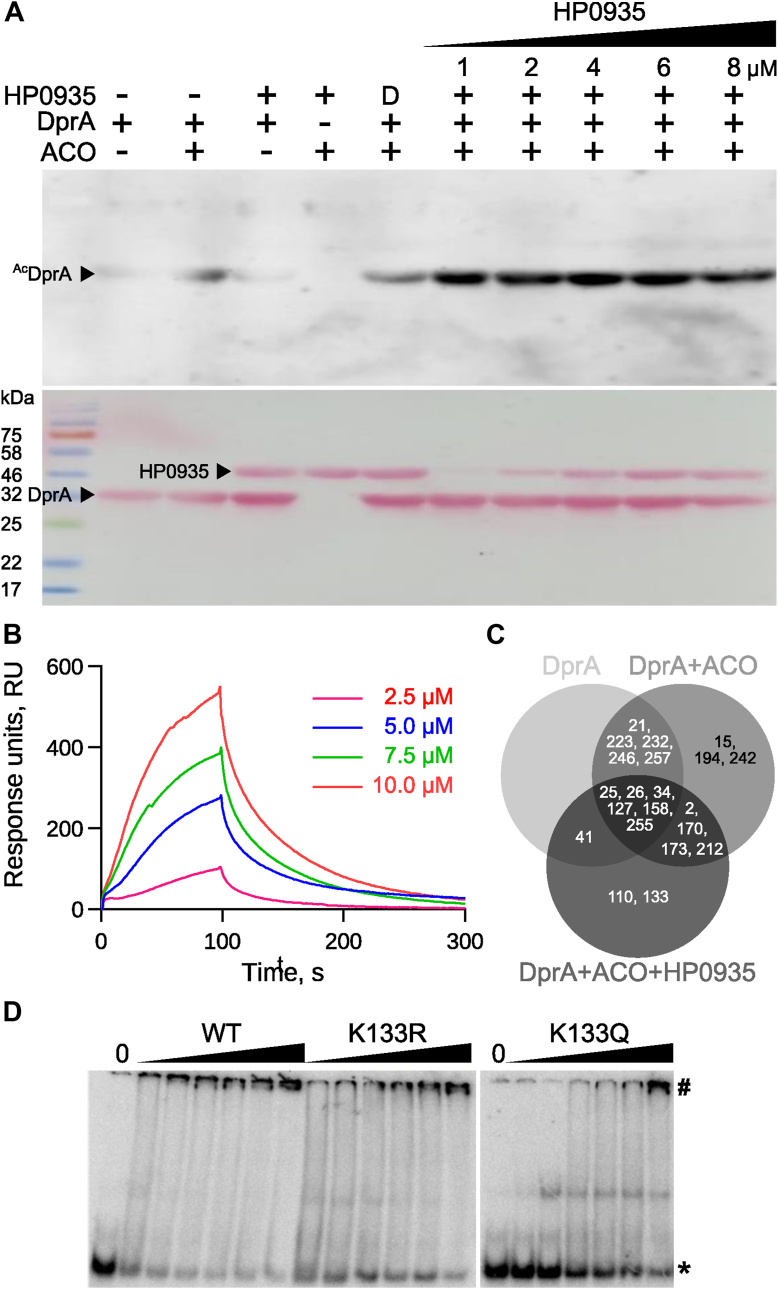
**Acetylation of DprA by HP0935.***A*, acetylation of *H. pylori* DprA with increasing concentration of GST-tagged HP0935 (1–8 μM). The reactions were separated on SDS-PAGE and acetylated DprA (^Ac^DprA) was probed by western blotting using anti-acetyl lysine antibodies (*top panel*). Ponceau stained blot showing protein loadings of GST-tagged HP0935 and DprA (*bottom panel*). *B*, interaction of HP0935 with DprA shown by surface plasmon resonance (SPR). The sensorgram is shown at different concentrations of HP0935. *C*, Venn diagram of acetylated lysine residues of DprA detected in acetylation reactions (R1: DprA, R2: DprA + ACO, or R3: DprA + ACO + HP0935) by mass spectrometry. *D*, DNA binding activity of DprA wild type (WT) and its point mutants (K133R, and K133Q) checked by electrophoretic mobility shift assay (EMSA). A 53-nucleotide single-stranded DNA (ssDNA) was used as a substrate. 0: control (no DprA), *asterisk* (∗): free ssDNA, and *hash* (#): nucleoprotein complex.

From Figure S1, it was clear that several proteins of *H. pylori* are prominently acetylated. Our laboratory has been working on structure-function relationships in several *H. pylori*'s proteins are involved in DNA repair, recombination, and natural transformation processes. Natural transformation is a tightly regulated, multistep process influenced by species-specific environmental conditions. It involves a series of proteins that facilitate DNA uptake and integration into the bacterial genome (39, 40, 41). Studies on *Streptococcus pneumoniae* suggest that DprA and RecA protect internalized ssDNA from bacterial nucleases. Inactivation of the *dprA* gene abolishes transformation (42). We previously demonstrated that *H. pylori* DprA facilitates natural transformation by binding to both ssDNA and dsDNA, acting as a physical barrier to nucleases, and stimulating DNA methylation to protect against the restriction-modification (R-M) barrier (43). However, the role of PTMs, such as acetylation, in natural transformation remains unexplored. To investigate this, the protein acetyltransferase activity of HP0935 against DprA (strain J99) as a substrate was probed by Western blotting using anti-acetyl lysine antibodies. Western blot analysis showed that HP0935 acetylates DprA in a concentration dependent mannar. However, minor non-enzymatic acetylation was observed due to ACO (Fig. 2*A*). To investigate the non-enzymatic acetylation of DprA, the protein was incubated with increasing concentrations of ACO and acetyl-phosphate. It was observed that both ACO and acetyl-phosphate acetylate DprA in a concentration-dependent manner. Further, surface plasmon resonance (SPR) spectroscopy confirmed a direct interaction between DprA and HP0935, with a dissociation constant (K_d_) of 3.28 μM (Fig. 2*B*). Mass spectrometry analysis of three acetylation reactions, R1 (only DprA), R2 (DprA + ACO), and R3 (DprA + ACO + HP0935), identified 19 out of a total of 24 lysine residues, with K110 and K133 uniquely acetylated in reaction R3 by HP0935 (Supporting Data 1, Fig. 2*C*). Notably, K133 in strain J99 corresponds to K137 in strain 26695, which interacts with ssDNA. A charge-reversal mutation (K137E) is known to reduce DNA binding activity (44). To assess the functional impact of K133 acetylation, two mutants were generated: K133R (charge-retaining) and K133Q (acetylation-mimicking). Electrophoretic mobility shift assay (EMSA) revealed that K133R retained WT-like DNA binding, whereas K133Q exhibited reduced activity (Fig. 2*D*). This suggests that K133 acetylation affects DprA’s ability to bind DNA, thereby potentially influencing *H. pylori* natural transformation. Given that protein acetylation serves regulatory roles in other bacteria, these findings highlight a possible role for acetylation in *H. pylori'*s genetic adaptability and transformation efficiency.

### Overall structure of apo- and ACO-HP0935

**Figure 3:**
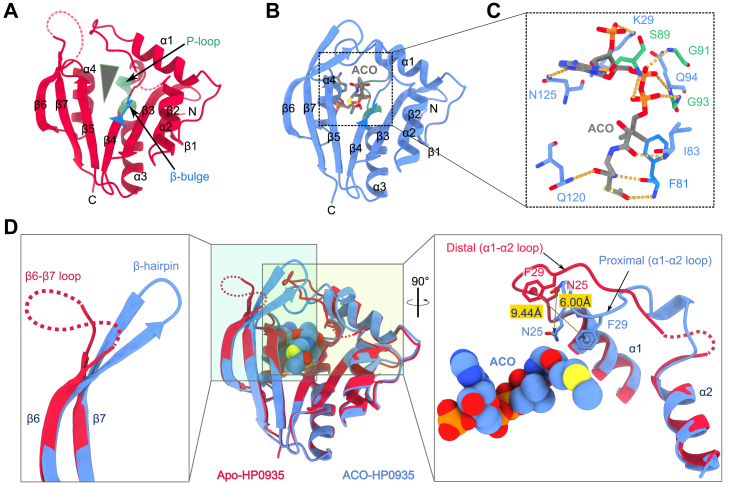
**Crystal structures of apo- and ACO-HP0935.***A*, Apo-HP0935 structure showing typical GNAT domain. The secondary structure elements are labeled: α1-α4 and β1-β7 (see Fig. S7*A*). The mixed β-sheet splayed between the β4 and β5 strands (indicated by a *grey triange*). The P-loop and the β-bulge are shown in *pale green* and *dodger blue* colors, respectively. *B*, ACO-HP0935 structure. ACO (*grey sticks*) binds in the V-shaped cleft *C*, interactions between HP0935 and ACO (see Fig. S8). *D*, conformational changes observed in α1-α2 and β6-β7 loops upon ACO binding. Superposed structures of apo- and ACO-bound HP0935 crystal structures (*middle panel*). ACO is rendered as spheres. In the ACO-HP0935 crystal structure, the α1-α2 loop undergoes a conformational transition where it is moved toward ACO. The α1-α2 loop conformation in apo-HP0935 structure is called distal as it is away from the ACO-binding cleft, whereas the conformation in ACO-HP0935 structure is called proximal as it is close to ACO. This conformational transition in the α1-α2 loop brings N25 and F29 close to ACO by 6.00 Å (distance between Cγ-atoms of N25) and 9.44 Å (distance between the phenyl ring centroids of F29), respectively, as shown by broken arrows (*right panel*). The disordered β6-β7 loop (*red broken line*) attains β6-β7 hairpin structure in ACO-bound structure (*left panel*). The missing residues of α1-α2 and β6-β7 loops in apo-HP0935 are shown as *broken lines* (*red*) in (*A* and *D*).

HP0935 exists as a monomer under physiological conditions (see Supporting information, Fig. S6, *A* and *B*). To gain structural insights, the crystal structures of HP0935 in its apo and ACO-bound forms were determined to be 2.00 Å and 2.40 Å resolution, respectively (see Experimental procedures, Supporting information, Table S2). The crystal structure of HP0935 revealed a conserved GNAT fold, characteristic of the GNAT superfamily (45, 46). The structure of HP0935 is composed of seven β-strands and four α-helices arranged in a topology—β1· α1· α2· β2· β3· β4· α3· β5· α4· β6· β7 (Figs. 3*A* and S7*A*). All β-strands form a central, twisted mixed β-sheet splayed between strands β4 and β5, which forms a wedge-shaped cleft for ACO and substrate binding. The four α-helices flank the β-sheet on both sides (Fig. 3*A*). Strand β4 contains a β-bulge structure that disrupts the β4 strand. This β-bulge structure is observed in many other GNAT family enzymes (45). The β-bulge can form, but not always, an oxyanion hole that stabilizes the tetrahedral reaction intermediate formed during catalysis (47, 48). Another feature of GNAT enzymes, the pyrophosphate-binding loop (P-loop), corresponding to the residues 88-QSQGLG-93 (Q-x-x-G-x-G) in HP0935, is located at a canonical position—a region spanning the c-terminal part of β4-α3 loop and the N-terminus of the α3 helix (Fig. 3*A*). ACO is bound in a tunnel formed by the α1-α2 loop, β4, β4-α3 loop, α3, β5, β5-α4 loop, and α4 (Fig. 3*B*). The conformation of ACO and its interactions are typical of other GNAT members (49). While the ACO is bound in a bent (L-shaped) conformation (Figs. 3*C* and S7*B*), the pyrophospha forms hydrogen bonds with residute moiety is accommodated by the P-loop, and the pantotheine moietyes on the β4 side of the wedge-shaped cleft (residues F81 and I83), mimicking the hydrogen bonding pattern of a parallel β-sheet structure. The adenine moiety of ACO is stabilized by hydrogen bonding with side chains of residues S89 and N125. The carbonyl oxygen of ACO’s acetyl group forms a hydrogen bond with the backbone amide (NH) of the β-bulge residue F81, which may stabilize the tetrahedral intermediate during the acetylation reaction (Figs. 3*C* and S8, *A* and *B*).

### Structural basis for ACO-induced conformational change in loops

Crystallization conditions A and B produced triangular-shaped apo-HP0935 crystals. However, in the presence of ACO, only condition B yielded crystals with an altered, needle-like, morphology (Experimental procedures, Fig. S9). This change in crystal morphology suggested a potential alteration in crystal packing, likely induced by structural changes in HP0935 upon ACO binding. To investigate this, the crystal structures of apo- and ACO-HP0935 were compared. The apo- and ACO-bound HP0935 crystallized in distinct space groups, C2 and P6_1_, respectively. A key observation was the partial disorder in the α1-α2 and complete disorder in the β6-β7 loop region in the apo-HP0935 structure, as evident from missing electron density. In contrast, these regions were well-ordered in the ACO-HP0935 structure, with a significant conformational change in the α1-α2 loop. In apo-HP0935, the α1-α2 loop adopts an extended, distal conformation, positioned away from the ACO-binding cleft. In contrast, in ACO-HP0935, the loop shifts toward ACO, adopting a proximal conformation (Fig. 3*D*). Despite a low overall Cα RMSD between the two structures (1.95 Å), the α1-α2 loop displayed high residue-wise RMSD values (Fig. S10), with residues N25 and F29 shifting towards ACO, by 6.00 Å and 9.44 Å, respectively (Fig. 3*D*). This transition constricts the ACO-binding cleft, likely stabilizing ACO and facilitating acetyl transfer. A similar observation has been reported in human Nα-acetyltransferase 60 (Naa60), where an F34A mutation in the α1-α2 loop abolished enzymatic activity (50).

Further, analysis of ACO/COA-bound structures revealed that a few GNAT members exhibited a similar distal-to-proximal loop transition upon ACO/COA binding (Table S3). In the aminoglycoside 6-N-acetyltransferase and the FdhC (sugar N-acetyltransferase), the distal α1-α2 loop is stabilized by crystal contacts in the apo state. However, in the presence of COA, the loop transitions to the proximal conformation and lacks crystal contacts (51, 52). In Rv0802c (acetyl- and succinyl-CoA transferase), the loop remains disordered in the apo state but adopts an ordered proximal conformation upon succinyl coenzyme A binding (53), suggesting ligand-induced stabilization. In HP0935, the α1-α2 loop is involved in crystal contacts in both conformations. In the distal conformation, two to three residues at the C-terminus of the loop are disordered, probably, due to the lack of stabilizing contacts. In contrast, in the proximal conformation, the loop is fully ordered and stabilized by interactions with the β6-β7 hairpin and symmetry-related molecules (Fig. S11, *A* and *B*). Given the distinct crystallization space groups and supporting evidence from the literature, it is likely that ACO binding induces the loop transition, which could further be stabilized by crystal packing. The effect of ACO on the distal conformation was further assessed using molecular dynamics simulations. The simulations showed that ACO affects the dynamics of the proximal conformation, and to some extent, the distal state explores a conformational space close to proximal conformation in the presence of ACO (see Supporting information, Fig. S12).

While the α1-α2 loop transition in HP0935 appears to be ACO-induced, the ordering of the β6-β7 loop is likely driven by crystal packing. In apo-HP0935, this region remains disordered, exposed to solvent channels, whereas in ACO-HP0935, it is ordered as a β-hairpin and forms part of a β-sheet alongside β1, β2, and β3 from a symmetry-related molecule. These findings suggest that the α1-α2 loop transition is primarily ACO-induced, possibly contributing to enzymatic activity. Ligand/substrate-induced conformational changes are widely recognized as critical for enzymatic function (54, 55).

### Acceptor substrate-binding region of HP0935

A structural similarity search using the DALI server (56) identified HP0935 structural homologs, including putative and small-molecule (57) acetyltransferases, as well as NATs (37, 58) and C-terminal lysine PAT (59). Despite low sequence conservation, structural similarities among GNAT family members are primarily due to the conserved core structure of GNAT fold (46). Structural differences among GNAT family members primarily stem from the α1-α2 loop, β6-β7 loop/hairpin, and the lengths and orientations of α1 and α2 helices—regions crucial for substrate binding (46, 49). These features define the structural and physicochemical properties of the substrate-binding region. However, no closely related structural homologs of HP0935 were found, with respect to its substrate-binding region.

Since HP0935 acetylates peptidyl Nε-lysine (Fig. 2*C*), its substrate-binding region was compared to those of known prokaryotic PAT structures. PATs targeting acetyl-coenzyme A synthetase (ACS) contain an additional α-helix in the α1-α2 loop, forming a narrow tunnel that accommodates only the peptidyl-lysine side chain (Fig. S13, *A*–*C*) (60, 61, 62). A similar architecture is present in C-terminal lysine PAT (Fig. S13*D*) (59). In the PatA-Acs complex, an extensive interaction interface is observed outside the substrate tunnel (60). Conversely, *E. coli* YajB has a shorter α1-α2 loop, with parallel α1 and α2 helices forming a narrow binding region (Fig. S13*E*) (63). Rv0802c, from *Mtb*, features a wider, cleft-like substrate-binding region within its tetrameric structure (Fig. S13*F*) (53, 64), whereas Eis from *Mtb* contains a substrate-binding channel between two domains (Fig. S13*G*) (33). In contrast, HP0935 exhibits a distinct substrate-binding region formed by an extended α1-α2 loop and β6-β7 hairpin that differs from other bacterial PATs (Fig. S13*H*).

**Figure 4:**
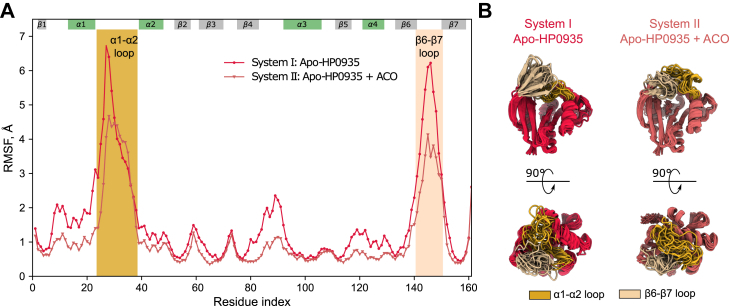
**Dynamics of α1-α2 and β6-β7 loops.***A*, backbone root mean square fluctuations (RMSF) of HP0935 simulation systems I and II. The vertical filled areas represent the α1-α2 loop (*golden yellow*) and β6-β7 loop (*peach puff*). The span of secondary structure elements (β-strand: *gray*, α-helix: *green*) along the residue index is shown at the *top* of the plot. *B*, representative structures from clustered simulation trajectories showing the widespread conformations of α1-α2 and β6-β7 loops.

The substrate-binding α1-α2 and β6-β7 loops undergo substrate-induced conformational changes (65, 66, 67). Root mean square fluctuation (RMSF) analysis of MD simulation data (systems I and II) revealed high flexibility in these loops, with a slight reduction in the presence of ACO (Fig. 4, *A* and *B*). These observations suggest that the conformational flexibility of these loops may contribute to the structural adaptability of the substrate-binding region, potentially facilitating HP0935’s ability to interact with diverse substrates. Loop dynamics influence the catalytic efficiency, specificity, and promiscuity of enzymes (66, 68, 69). Given that HP0935 is the only known GNAT protein acetyltransferase present in *H. pylori*, and the prevalence of protein lysine acetylation in this organism (Fig. S1), we hypothesize that the observed loop flexibility may enable HP0935 to acetylate multiple substrates, potentially influencing bacterial physiology. However, the role of loop dynamics in the broad-substrate specificity of HP0935 remains to be evaluated experimentally.

### Catalytic mechanism

**Figure 5:**
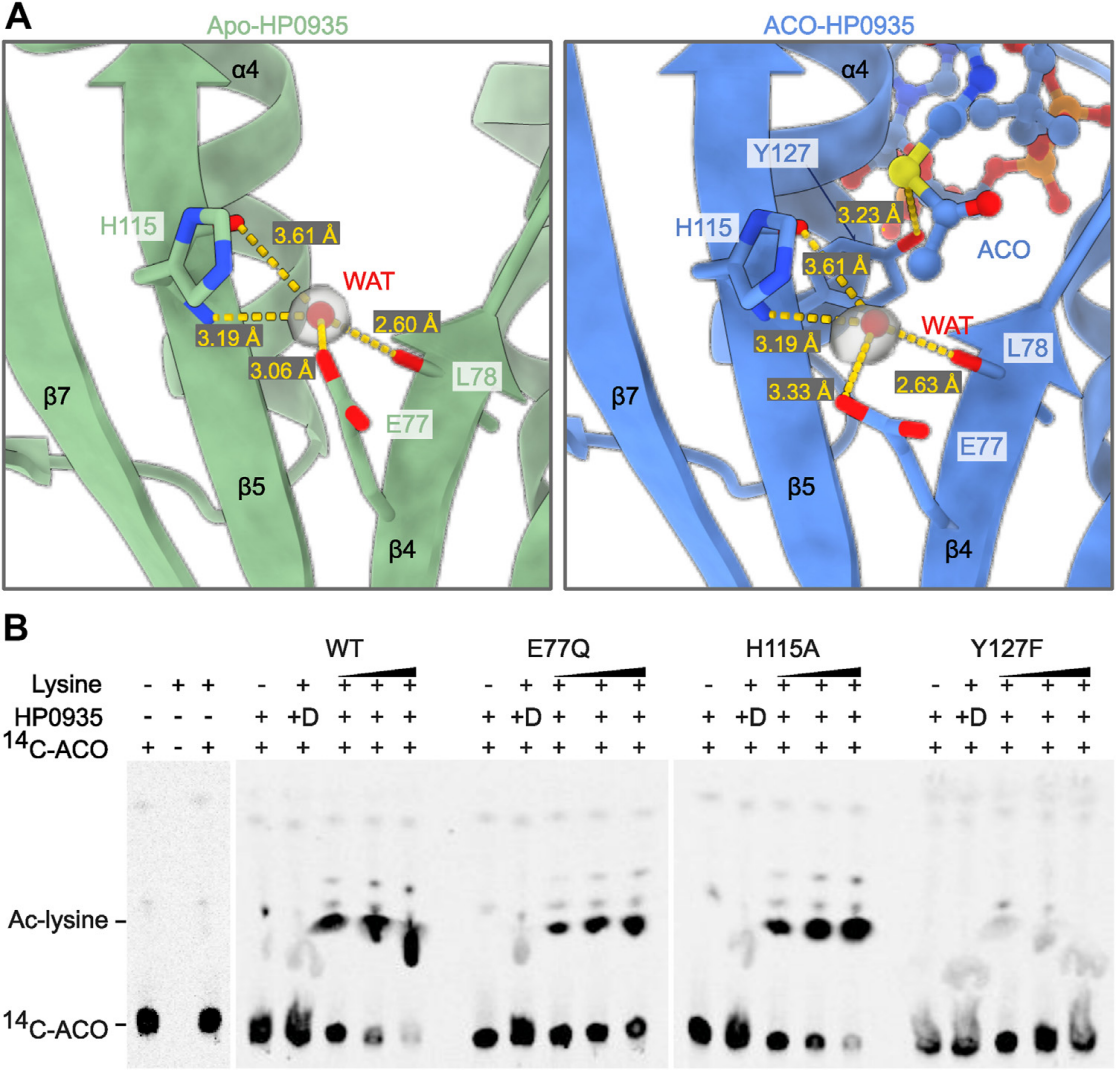
**The active site of HP0935.***A*, HP0935 contains two putative general base residues (E77 and H115) and a general acid (Y127) at the catalytic site. A conserved water molecule (WAT) is shown for apo- and ACO-HP0935 crystal structures at the catalytic site. This water molecule is coordinated with L78 and H115 backbone atoms, and the E77 side chain by hydrogen bonding. The electron density (2F_o_ - F_c_) map, contoured at 1.5 σ level, is shown for the conserved water molecule. *B*, acetyltransferase activity of HP0935 wild type (WT) and its variants (E77Q, H115A, and Y127F) was tested with lysine as a substrate. The acetylation reaction was separated by silica gel TLC and imaged by autoradiography. ^14^C-ACO: ^14^C labeled acetyl-coenzyme A, Ac-lysine: acetylated lysine, and D: heat-denatured HP0935.

Acetylation of an amino acid or a protein requires deprotonation of the Nα/Nε-amino group before a nucleophilic attack on the acetyl group of ACO. Previous studies have shown that the deprotonation of the amino group is facilitated by a conserved general base residue(s) or a conserved catalytic water molecule, while a general acid protonates the leaving group, COA-S^−^ (45). Structural analysis of HP0935 identified E77 and H115 as potential general base residues and Y127 as a general acid. A conserved water molecule, hydrogen-bonded to L78, H115, and E77, was observed (Fig. 5*A*).

The role of these putative catalytic residues was investigated using lysine as a substrate and HP0935 point mutants: E77Q, H115A, and Y127F. Circular dichroism confirmed the structural integrity of these point mutants (data not shown). Activity assays showed a slight reduction in activity for the E77Q mutant, while H115A retained WT-like activity. A similar observation was made for *E. coli* PatZ where an E809 point mutation showed only a 20% reduction in activity (70). In the case of yeast GCN5 acetyltransferase, the general base is indispensable (71). In contrast, Y127F showed negligible activity (Fig. 5*B*). This suggests that for lysine substrate, the general base is not essential for acetylation, likely due to the following reasons: at assay pH 8.0, approximately 10% of lysine’s Nα-amino group exists in a deprotonated state which serves as a substrate and the equilibrium maintains the deprotonated state of lysine during acetylation. Alternatively, HP0935 could utilize a conserved water molecule for lysine deprotonation. C-terminal lysine protein acetyltransferase does not contain a general base residue at the active site; however, a conserved water molecule present at the active site was proposed to do this job (59).

In summary, we identified and cloned a putative acetyltransferase gene, *hp0935*. The purified HP0935 protein was active against amino acids and protein substrate. We showed that HP0935 acetylates DprA, *in vitro*, thus regulating its DNA-binding properties, and hence possibly natural transformation. The 3D structure of HP0935 was determined in the apo- and ACO-bound forms. Molecular dynamics simulation data showed that the loops at the substrate binding region are highly dynamic. The conformational flexibility of these loops is likely to facilitate the acetylation of multiple substrates by an induced fit, conformational selection, or a combination of both mechanisms. In conclusion, this study clearly shows that HP0935 is likely the only protein acetyltransferase that acetylates free amino acids (Nα and Nε) and protein substrates (Nε-lysine), highlighting the robustness of acetylation in the human pathogen *H. pylori*.

## Experimental procedures

### Identification of putative N-acetyltransferase

A putative N-acetyltransferase from *H. pylori* was identified using PSI-BLAST (http://www.ncbi.nlm.nih.gov/BLAST/). The GNAT domain of known protein acetyltransferases from organisms *S. enterica, S. cerevisiae, M. smegmatis,* and *M. tuberculosis* were used as queries against the “non-redundant protein sequences (nr)” database. Hits from the first round of searches with an e-value <0.005 were included in the second iteration. Each query sequence underwent six iterations in total.

### Cloning and mutagenesis

The gene sequence of *hp0935* was procured from the annotated whole-genome sequence of *H. pylori* 26695 strain from JCVI (J. Craig Venter Institute). The gene was amplified from the genomic DNA of *H. pylori* 26695 strain by polymerase chain reaction (PCR) using primers HP0935-His-FP and HP0935-His-RF for His-tagged HP0935 and primers HP0935-GST-FP and HP0935-GST-RP for GST-tagged HP0935 (Table S4). The restriction enzyme-digested PCR products were ligated into the pET15b and pGEX_4T2 vectors linearized with the same enzymes, generating the recombinant DNA constructs pET15b-*hp0935* and pGEX_4T2-*hp0935*, respectively. For crystallization purposes, the hp0935 gene was amplified from the pET15b-*hp0935* construct and cloned into pNIC28-Bsa4 plasmid by ligation-independent cloning to generate the pNIC28-*hp0935* construct.

Site-directed mutagenesis, a technique described earlier (72), was used to replace the amino acid of interest in the protein. To replace the amino acid E77 with glutamine, H115 with alanine, and Y127 with phenylalanine in HP0935 protein, sense and antisense primers (Table S4) were used against the template pGEX_4T2-*hp0935* clone for PCR. Q5 polymerase was used for the PCR amplification of the gene. The mutagenic primers introduced a suitable restriction enzyme recognition site. The mutations were further confirmed by DNA sequencing.

### Peptide mass fingerprinting

Peptide mass fingerprint analysis of trypsin-treated HP0935 was carried out as described (73). MALDI-MS data were acquired on an ultraflex TOF/TOF spectrometer (Bruker Daltonics and Bremen) equipped with 50 Hz pulsed nitrogen laser (11/4337 nm), operated in positive ion reflection mode using a 90-ns time delay, and a 25 kV accelerating voltage. Samples were prepared by mixing an equal volume of peptide (0.5 ml) with matrices dihydroxybenzoic acid/α-cyano-4-hydroxycinnamic acid saturated in 0.1% trifluoroacetic acid and acetonitrile (1:1 v/v). Masses below 500 m/z were not considered because of interference from the matrix. Using Mascot software, peptide mass fingerprints obtained by MALDI-TOF MS were used to search NCBInr, SWISS-PROT, and MSDB databases (http://www.matrixscience.com). The parameters used for the search were as follows: peptide mass ranged from 800 to 3500 U; modifications were allowed for carboxyamidomethylation of cysteine and oxidation of methionine; one missed cleavage site was allowed; mass accuracy was 1 U; restriction was placed on Bacteria (Eubacteria). Top-scoring hits were analyzed for sequence coverage (>50%) and number of matched peptides.

### Acetylation assays

Acetyltransferase activity of WT and mutant HP0935 was assayed with amino acids (methionine, lysine, arginine, or proline), acetylated lysine (Nα-acetyl lysine or Nε-acetyl lysine), and small peptides as substrates. A reaction mixture (10 μl) containing 12 mM substrate, ^14^C-ACO (^14^C-labeled acetyl-coenzyme A), and 2 μM HP0935 in an assay buffer (50 mM Tris-HCl, pH 8.0, 50 mM NaCl) was incubated at 30 °C for 2 h, and the products were resolved by silica gel TLC using butanol, formic acid, and water mixture (5:3:1) as mobile phase. TLC silica plates were dried, exposed overnight to a Phosphor-imaging cassette, and imaged using Fujifilm reader. Acetylation products were quantified by densitometry using IMAGEGAUGE V2.54 software (Fujifilm).

To determine the kinetic parameters for lysine, 800 nM HP0935 was incubated with 50 μM ^14^C-ACO and varying concentrations of lysine (1–50 mM) in an assay buffer (50 mM Tris-HCl, pH 8.0, 50 mM NaCl) at 30 °C for 20 min. The reaction mixture was resolved, and the products were quantified as described above. Similarly, kinetic parameters for ACO were determined similarly at 50 mM lysine and varying [^14^C]-ACO concentrations (5–50 μM). The data were fit to Michaelis-Menten equation.

*In vitro* acetylation of DprA was carried out in 50 mM Tris-Cl (pH 8.0) buffer containing 50 mM NaCl, 200 μM ACO, and 4 μM DprA at 30 °C for 2 h. The reaction was initiated by the addition of an increasing concentration of HP0935 (1–8 μM). Acetylated reactions were separated on 0.1% SDS - 10% PAGE and transferred onto the nitrocellulose membrane. The membrane was blocked with 5% w/v skimmed milk for 2 h at room temperature followed by incubation in 5% BSA in PBST (phosphate-buffered saline with 0.05% Tween 20) containing anti-acetyl lysine antibody (1:1000 dilution) from Abcam at 4 °C for overnight. The blot was washed with PBST and further incubated in PBST containing HRP-conjugated anti-rabbit antibody (1:10,000 dilution). The blots were then developed with an ECL kit (Merck Life Science) using a LAS3000 image analyzer.

### *In vitro* acetylation of DprA and mass spectrometry analysis

All three *in vitro* acetylation reactions were set up in a reaction buffer containing 50 mM Tris-Cl (pH 8.0) and 50 mM NaCl: (i) unacetylation control (R1: DprA only), (ii) non-enzymatic acetylation (R2: DprA + 200 μM ACO), and (iii) enzymatic acetylation (R3: DprA + 200 μM ACO + 8 μM HP0935). All reactions were incubated at 30 °C for 2 h. Recombinant His-tagged DprA (R1 – R3) and GST-tagged HP0935 (R3) proteins purified from *E. coli* were used. DprA from these reactions was purified using Ni-NTA resin. The binding of DprA to Ni-NTA resin was performed in reaction buffer (50 mM Tris-HCl (pH 8.0), 50 mM NaCl and 200 μM ACO). The slurry was washed with the reaction buffer containing 10 mM imidazole. His-tag DprA was eluted from Ni-NTA resin using the reaction buffer containing 300 mM imidazole. Purified protein was dialyzed in 50 mM (NH_4_)HCO_3_ for 8 h at 4 °C. DprA protein was digested with mass spectrometry-grade trypsin (NEB #8101S) at a 1:50 ratio (w/w) for 16 h at 37 °C/300 RPM. The digested peptide was purified using C18 resin Zip/Tip. Purified peptides were analyzed by LC-MS/MS using a Q Exactive Plus mass spectrometer (Thermo Scientific) coupled with a nano-HPLC system. Peptides were separated on a nano-HPLC column over a 100-min run time. The mobile phase consisted of A: 0.1% formic acid in water, and B: 0.1% formic acid in acetonitrile. A gradient of 25% B was achieved over 100 min at a flow rate of 300 nl/min. Mass spectrometry data were acquired with an MS1 resolution of 70,000 and an MS2 resolution of 17,500. Peptide quantification was performed using a Tandem Mass Tag (TMT). Raw mass spectrometry data were processed using Proteome Discoverer 2.1 (Thermo Scientific) to generate peak lists. Peptides were identified against a FASTA database derived from the *H. pylori* 26695 strain proteome. Search parameters included fixed modification of carbamidomethylation and variable modification of oxidation, with up to two missed cleavages allowed due to trypsin specificity. Precursor and product ion mass tolerances were set to 1.2 Da. A false discovery rate (FDR) of 0.05 was applied to filter peptide identifications, ensuring high-confidence results. The analysis identified 45 unique and razor peptides, reflecting the protein’s acetylation profile. The experiment was conducted with two biological replicates and two technical replicates. The data were provided in Supporting Data 1 (Excel file).

### NMR spectroscopy

Samples for NMR spectroscopy were prepared by aliquoting 600 μl of the reaction products (*vide supra*) and adding 60 μl of D2O (99.9%) as the lock solvent. All NMR spectra were acquired on a Bruker 700 MHz NEO NMR spectrometer equipped with a room temperature triple resonance probe fitted with a single (z-axis only) pulse field gradient accessory. NMR spectra were acquired at 25 °C. One-dimensional NMR spectra were acquired using the excitation sculpting pulse scheme for solvent suppression. A total of 8192 complex points were sampled over a spectral width of 9000 Hz. Spectra were exponentially multiplied with a line broadening parameter of 5 Hz, Fourier transformed, and analyzed using Topspin 4.0.9 software.

### Electrophoretic mobility shift assays

The DNA-binding activity of DprA was estimated in 1X TAM reaction buffer (50 mM Tris-Cl pH 7.4, 50 mM sodium acetate, 10 mM magnesium acetate, and 1 mM dithiothreitol (DTT)) containing 1 nM of 32P labeled ssDNA (53Forward, Table. S4). An increasing concentration of DprA was added to start the reaction (100–1000 nM) in a 20 μl reaction volume. The reaction was then incubated at 4 °C for 30 min, and the reaction was resolved on 8% native-PAGE in 1X TAME buffer (6 mM Tris-Cl (pH 7.8), 10 mM sodium acetate, 4 mM magnesium acetate, and 1 mM ethylenediaminetetraacetic acid (EDTA)) at 100 V for 8 h, 4 °C. The gel was vacuum dried at 80 °C for 45 min after transferring onto Whatman 3 mm paper and imaged using Phospho-Imager (Fuji FLA-9000).

### Surface plasmon resonance

The protein-protein interaction of DprA and HP0935 was investigated by surface plasmon resonance (Biacore3000 from GE Healthcare Life Science) as described earlier (43). Briefly, the DprA protein was immobilized on the surface of the CM5 sensor chip (GE Healthcare Life Science). Individual proteins’ interaction with the CM5 chip surface resulted in a ∼500 RU/flow cell response signal. One of the flow cells was treated with ethanolamine alone to score the non-specific binding. An interaction study was performed in 50 mM Tris-Cl (pH7.4) buffer at 25 °C with a flow rate of 20 μl/min. Varying concentrations of HP0935 were passed over the flow cell surface, followed by a dissociation period of 300 s. The regeneration of the flow cell surface was performed by 50 mM NaOH. The blank reference flow cell corrected all the protein-protein interaction data for non-specific binding. Each experiment was repeated twice to ensure reproducibility. The standard Langmuir binding equation was a good fit for the association and dissociation phases.

### Crystallization

Crystallization trials were carried out with tag-removed HP0935 at 22 °C using the sitting-drop vapor diffusion method. We were able to grow apo-form HP0935 crystals in two different crystallization conditions (condition A: 0.2 M potassium thiocyanate, 0.1 M Bis-Tris propane pH 6.5, and 20% (w/v) polyethylene glycol 3500 and condition B: 0.02 M magnesium chloride hexahydrate, 0.1 M HEPES pH 7.5 and 22% (w/v) poly(acrylic acid sodium salt) 5100) with identical morphology (triangular plate-shaped, Fig. S9, *A* and *B*). Final diffraction quality crystals were obtained at 4 °C in a crystallization drop set up with 1 μl of protein (5 mg/ml) solution and 1 μl of reservoir solution in 2 days. For growing cofactor-bound (ACO) crystals, protein at 16 mg/ml concentration was incubated with five-fold molar excesses of ACO on ice for 1 h. Crystallization was carried out at 4 °C similarly as described above. Needle-like crystals were obtained exclusively in condition B (Fig. S9*C*).

### Data collection and structure solution

Anomalous datasets were collected on apo-protein crystals and native datasets were collected on apo- and ACO-bound-protein crystals. To collect anomalous data, crystals were soaked in a reservoir solution containing 400 mM of sodium iodide for 10 min. Both anomalous and native data sets were collected (1° slices covering 360°) at a home source (Bruker Microstar, Cu Kα λ = 1.5418 Å). Diffraction data for ACO-bound protein crystals were collected at ID29 (ESRF, Grenoble) at a wavelength of 1.0723 Å, with 0.1° slices covering 360°. Prior to data collection, all the crystals were cryo-protected with 20% (v/v) ethylene glycol and flash-frozen in liquid nitrogen. All datasets were collected at 100 K. Anomalous and native datasets for apo crystals were processed using Burker’s PROTEUM3 suite, to resolutions of 2.42 Å and 2.00 Å, respectively. The ACO-bound dataset was processed using XDSAPP3.0 (74) to 2.40 Å resolution. Experimental phases were determined from the anomalous dataset using the iodine SAD phasing method with the CRANK2 pipeline (75). A partial model obtained from CRANK2 was built to completion using Buccaneer (76). High-resolution structures of both apo and ACO-bound forms were determined from the native datasets by molecular replacement (MR) method using Phaser-MR (77). Model building was completed by iterative manual building with Coot (78) and refinement with REFMAC5 (79). Model quality was assessed using MolProbity (80).

### Miscellaneous

All data plots were generated using GraphPad Prism and Python plotting library, Matplotlib (81). The structures and trajectories were visualized with ChimeraX (82). All artwork was prepared using Inkscape (https://inkscape.org).

### Statistical analysis

All enzyme kinetic data analyses, including both linear and non-linear regressions, were performed with GraphPad Prism version 8.0. Lineweaver-Burke plot linear regressions for all enzyme kinetic data have a *p*-value <0.005. Unless otherwise noted, all enzyme activity data were the average of at least duplicate determinations. The error bars represent the standard deviation (SD).

## Data availability

The coordinates for apo-HP0935 and ACO-HP0935 structures have been deposited in the Protein Data Bank under the accession codes 8IYM and 8IYO, respectively.

## Supporting information

This article contains supporting information (43, 45, 78, 83, 84, 85, 86, 87, 88, 89, 90, 91, 92, 93, 94).

## Conflict of interest

The authors declare that they have no conflicts of interest with the contents of this article.

## References

[1] Verdone L., Agricola E., Caserta M., Di Mauro E. (2006). Histone acetylation in gene regulation. Brief. Funct. Genomic. Proteomic..

[2] Wang Q., Zhang Y., Yang C., Xiong H., Lin Y., Yao J. (1979). (2010) Acetylation of metabolic enzymes coordinates carbon source utilization and metabolic flux. Science.

[3] Guan K.L., Xiong Y. (2011). Regulation of intermediary metabolism by protein acetylation. Trends Biochem. Sci..

[4] van de Kooij B., de Vries E., Rooswinkel R.W., Janssen G.M.C., Kok F.K., van Veelen P.A. (2023). N-terminal acetylation can stabilize proteins independent of their ubiquitination. Sci. Rep..

[5] Choudhary C., Kumar C., Gnad F., Nielsen M.L., Rehman M., Walther T.C. (2009). Lysine acetylation targets protein complexes and co-regulates major cellular functions. Science.

[6] Starai V.J., Escalante-Semerena J.C. (2004). Identification of the protein acetyltransferase (Pat) enzyme that acetylates acetyl-CoA synthetase in Salmonella enterica. J. Mol. Biol..

[7] Ren J., Sang Y., Lu J., Yao Y.F. (2017). Protein acetylation and its role in bacterial virulence. Trends Microbiol..

[8] Drazic A., Myklebust L.M., Ree R., Arnesen T. (2016). The world of protein acetylation. Biochim. Biophys. Acta.

[9] Tanner K.G., Langer M.R., Kim Y., Denu J.M. (2000). Kinetic mechanism of the histone acetyltransferase GCN5 from yeast. J. Biol. Chem..

[10] Hentchel K.L., Escalante-Semerena J.C. (2015). Acylation of biomolecules in prokaryotes: a widespread strategy for the control of biological function and metabolic stress. Microbiol. Mol. Biol. Rev..

[11] VanDrisse C.M., Escalante-Semerena J.C. (2019). Protein acetylation in bacteria. Annu. Rev. Microbiol..

[12] Marshall B., Warren J.R. (1984). Unidentified curved bacilli in the stomach of patients with gastritis and peptic ulceration. Lancet.

[13] Kusters J.G., Van Vliet A.H.M., Kuipers E.J. (2006). Pathogenesis of Helicobacter pylori infection. Clin. Microbiol. Rev..

[14] Hooi J.K.Y., Lai W.Y., Ng W.K., Suen M.M.Y., Underwood F.E., Tanyingoh D. (2017). Global prevalence of *Helicobacter pylori* infection: systematic review and meta-analysis. Gastroenterology.

[15] Lee Y.C., Dore M.P., Graham D.Y. (2022). Diagnosis and treatment of *Helicobacter pylori* infection. Annu. Rev. Med..

[16] Blaser M.J., Berg D.E. (2001). *Helicobacter pylori* genetic diversity and risk of human disease. J. Clin. Invest..

[17] Celli J.P., Turner B.S., Afdhal N.H., Keates S., Ghiran I., Kelly C.P. (2009). *Helicobacter pylori* moves through mucus by reducing mucin viscoelasticity. Proc. Natl. Acad. Sci. U. S. A..

[18] Kersulyte D., Chalkauskas H., Berg D.E. (1999). Emergence of recombinant strains of *Helicobacter pylori* during human infection. Mol. Microbiol..

[19] Krisch L.M., Posselt G., Hammerl P., Wesslera S. (2016). CagA phosphorylation in *Helicobacter pylori*-infected B cells is mediated by the nonreceptor tyrosine kinases of the Src and Abl families. Infect. Immun..

[20] Lock R.A., Cordwell S.J., Coombs G.W., Walsh B.J., Forbes G.M. (2001). Proteome analysis of *Helicobacter pylori*: major proteins of type strain NCTC 11637. Pathology.

[21] Tejada-Arranz A., Lulla A., Bouilloux-Lafont M., Turlin E., Pei X.Y., Douché T. (2023). Acetylation regulates the oligomerization state and activity of RNase J, the *Helicobacter pylori* major ribonuclease. Nat. Commun..

[22] Pan J., Ye Z., Cheng Z., Peng X., Wen L., Zhao F. (2014). Systematic analysis of the lysine acetylome in *Vibrio parahemolyticus*. J. Proteome Res..

[23] Liu F., Yang M., Wang X., Yang S., Gu J., Zhou J. (2014). Acetylome analysis reveals diverse functions of lysine acetylation in *Mycobacterium tuberculosis*. Mol. Cell Proteomics.

[24] Liu L., Wang G., Song L., Lv B., Liang W. (2016). Acetylome analysis reveals the involvement of lysine acetylation in biosynthesis of antibiotics in *Bacillus amyloliquefaciens*. Sci. Rep..

[25] Li L., Wang W., Zhang R., Xu J., Wang R., Wang L. (2018). First acetyl-proteome profiling of Salmonella Typhimurium revealed involvement of lysine acetylation in drug resistance. Vet. Microbiol..

[26] Quevillon E., Silventoinen V., Pillai S., Harte N., Mulder N., Apweiler R. (2005). InterProScan: protein domains identifier. Nucleic Acids Res..

[27] Schoenhofen I.C., McNally D.J., Brisson J.R., Logan S.M. (2006). Elucidation of the CMP-pseudaminic acid pathway in *Helicobacter pylori*: synthesis from UDP-N-acetylglucosamine by a single enzymatic reaction. Glycobiology.

[28] Ma X., Jiang K., Zhou C., Xue Y., Ma Y. (2022). Identification and characterization of a novel GNAT superfamily Nα-acetyltransferase from *Salinicoccus halodurans* H3B36. Microb. Biotechnol..

[29] Bode R., Thurau A.M., Schmidt H. (1993). Characterization of acetyl-CoA: l-lysine N6-acetyltransferase, which catalyses the first step of carbon catabolism from lysine in *Saccharomyces cerevisiae*. Arch. Microbiol..

[30] Bienvenut W.V., Brünje A., Boyer J., Mühlenbeck J.S., Bernal G., Lassowskat I. (2020). Dual lysine and N-terminal acetyltransferases reveal the complexity underpinning protein acetylation. Mol. Syst. Biol..

[31] Radkov A.D., Moe L.A. (2014). Bacterial synthesis of D-amino acids. Appl. Microbiol. Biotechnol..

[32] Yow G.Y., Uo T., Yoshimura T., Esaki N. (2004). D-Amino acid-N-acetyltransferase of *Saccharomyces cerevisiae*: a close homologue of histone acetyltransferase Hpa2p acting exclusively on free D-amino acids. Arch. Microbiol..

[33] Kim K.H., An D.R., Song J., Yoon J.Y., Kim H.S., Yoon H.J. (2012). *Mycobacterium tuberculosis* Eis protein initiates suppression of host immune responses by acetylation of DUSP16/MKP-7. Proc. Natl. Acad. Sci. U. S. A..

[34] Pan Q., Zhao F.L., Ye B.C. (2018). Eis, a novel family of arylalkylamine N-acetyltransferase (EC 2.3.1.87). Sci. Rep..

[35] Ghosh S., Padmanabhan B., Anand C., Nagaraja V. (2016). Lysine acetylation of the *Mycobacterium tuberculosis* HU protein modulates its DNA binding and genome organization. Mol. Microbiol..

[36] Sampath V., Liu B., Tafrov S., Srinivasan M., Rieger R., Chen E.I. (2013). Biochemical characterization of Hpa2 and Hpa3, two small closely related acetyltransferases from *Saccharomyces cerevisiae*. J. Biol. Chem..

[37] Vetting M.W., Bareich D.C., Yu M., Blanchard J.S. (2008). Crystal structure of RimI from *Salmonella typhimurium* LT2, the GNAT responsible for Nα-acetylation of ribosomal protein S18. Protein Sci..

[38] Deng S., Marmorstein R. (2021). Protein N-terminal acetylation: structural basis, mechanism, versatility, and regulation. Trends Biochem. Sci..

[39] Fontaine L., Wahl A., Fléchard M., Mignolet J., Hols P. (2015). Regulation of competence for natural transformation in streptococci. Infect. Genet. Evol..

[40] Chen I., Dubnau D. (2004). DNA uptake during bacterial transformation. Nat. Rev. Microbiol..

[41] Johnston C., Martin B., Fichant G., Polard P., Claverys J.P. (2014). Bacterial transformation: distribution, shared mechanisms and divergent control. Nat. Rev. Microbiol..

[42] Bergé M., Mortier-Barrière I., Martin B., Claverys J.P. (2003). Transformation of *Streptococcus pneumoniae* relies on DprA- and RecA-dependent protection of incoming DNA single strands. Mol. Microbiol..

[43] Dwivedi G.R., Sharma E., Rao D.N. (2013). *Helicobacter pylori* DprA alleviates restriction barrier for incoming DNA. Nucleic Acids Res..

[44] Wang W., Ding J., Zhang Y., Hu Y., Wang D.C. (2014). Structural insights into the unique single-stranded DNA-binding mode of *Helicobacter pylori* DprA. Nucleic Acids Res..

[45] Salah Ud-Din A.I., Tikhomirova A., Roujeinikova A. (2016). Structure and functional diversity of GCN5-related N-acetyltransferases (GNAT). Int. J. Mol. Sci..

[46] Vetting M.W., Luiz L.P., Yu M., Hegde S.S., Magnet S., Roderick S.L. (2005). Structure and functions of the GNAT superfamily of acetyltransferases. Arch. Biochem. Biophys..

[47] Bhatnagar R.S., Fütterer K., Farazi T.A., Korolev S., Murray C.L., Jackson-Machelski E. (1998). Structure of N-myristoyltransferase with bound myristoylCoA and peptide substrate analogs. Nat. Struct. Biol..

[48] Farazi T.A., Waksman G., Gordon J.I. (2001). Structures of *Saccharomyces cerevisiae* N-myristoyltransferase with bound myristoyl CoA and peptide provide insights about substrate recognition and catalysis. Biochemistry.

[49] Dyda F., Klein D.C., Hickman A.B. (2003). GCN5-related N-acetyltransferases: a structural overview. Annu. Rev. Biophys. Biomol. Struct..

[50] Chen J.Y., Liu L., Cao C.L., Li M.J., Tan K., Yang X. (2016). Structure and function of human Naa60 (NatF), a Golgi-localized bi-functional acetyltransferase. Sci. Rep..

[51] Maurice F., Broutin I., Podglajen I., Benas P., Collatz E., Dardel F. (2008). Enzyme structural plasticity and the emergence of broad-spectrum antibiotic resistance. EMBO Rep..

[52] Salinger A.J., Thoden J.B., Holden H.M. (2016). Structural and functional investigation of FdhC from *Acinetobacter nosocomialis*: a sugar N-acyltransferase belonging to the GNAT superfamily. Biochemistry.

[53] Vetting M.W., Errey J.C., Blanchard J.S. (2008). Rv0802c from *Mycobacterium tuberculosis*: the first structure of a succinyltransferase with the GNAT fold. Acta Crystallogr. Sect. F Struct. Biol. Cryst. Commun..

[54] Cheng X., Shaltiel S., Taylor S.S. (1998). Mapping substrate-induced conformational changes in cAMP-dependent protein kinase by protein footprinting. Biochemistry.

[55] Eginton C., Naganathan S., Beckett D. (2015). Sequence–function relationships in folding upon binding. Protein Sci..

[56] Holm L. (2020). DALI and the persistence of protein shape. Protein Sci..

[57] Filippova E.V., Shuvalova L., Minasov G., Kiryukhina O., Zhang Y., Clancy S. (2011). Crystal structure of the novel PaiA N-acetyltransferase from *Thermoplasma acidophilium* involved in the negative control of sporulation and degradative enzyme production. Proteins.

[58] Ma C., Pathak C., Jang S., Lee S.J., Nam M., Kim S.J. (2014). Structure of *Thermoplasma volcanium* Ard1 belongs to N-acetyltransferase family member suggesting multiple ligand binding modes with acetyl coenzyme A and coenzyme A. Biochim. Biophys. Acta.

[59] Majorek K.A., Kuhn M.L., Chruszcz M., Anderson W.F., Minor W. (2013). Structural, functional, and inhibition studies of a Gcn5-related N-acetyltransferase (GNAT) superfamily protein PA4794: a new C-terminal lysine acetyltransferase from *Pseudomonas aeruginosa*. J. Biol. Chem..

[60] Tucker A.C., Taylor K.C., Rank K.C., Rayment I., Escalante-Semerena J.C. (2014). Insights into the specificity of lysine acetyltransferases. J. Biol. Chem..

[61] Lee H.J., Lang P.T., Fortune S.M., Sassetti C.M., Alber T. (2012). Cyclic AMP regulation of protein lysine acetylation in *Mycobacterium tuberculosis*. Nat. Struct. Mol. Biol..

[62] Brent M.M., Iwata A., Carten J., Zhao K., Marmorstein R. (2009). Structure and biochemical characterization of protein acetyltransferase from *Sulfolobus solfataricus*. J. Biol. Chem..

[63] Lu J., Wang X., Xia B., Jin C. (2009). Solution structure of apo-YjaB from *Escherichia coli*. Proteins.

[64] Anand C., Santoshi M., Singh P.R., Nagaraja V. (2021). Rv0802c is an acyltransferase that succinylates and acetylates *Mycobacterium tuberculosis* nucleoid-associated protein HU. Microbiology.

[65] Tanner K.G., Trievel R.C., Kuo M.H., Howard R.M., Berger S.L., Allis C.D. (1999). Catalytic mechanism and function of invariant glutamic acid 173 from the histone acetyltransferase GCN5 transcriptional coactivator. J. Biol. Chem..

[66] Abboud A., Bédoucha P., Byška J., Arnesen T., Reuter N. (2020). Dynamics-function relationship in the catalytic domains of N-terminal acetyltransferases. Comput. Struct. Biotechnol. J..

[67] Weidenhausen J., Kopp J., Armbruster L., Wirtz M., Lapouge K., Sinning I. (2021). Structural and functional characterization of the N-terminal acetyltransferase Naa50. Structure.

[68] Corbella M., Pinto G.P., Kamerlin S.C.L. (2023). Loop dynamics and the evolution of enzyme activity. Nat. Rev. Chemistry..

[69] Dhindwal S., Patil D.N., Mohammadi M., Sylvestre M., Tomar S., Kumar P. (2011). Biochemical studies and ligand-bound structures of biphenyl dehydrogenase from *Pandoraea pnomenusa* strain B-356 reveal a basis for broad specificity of the enzyme. J. Biol. Chem..

[70] De Diego Puente T., Gallego-Jara J., Castaño-Cerezo S., Sánchez V.B., Espín V.F., De La Torre J.G. (2015). The protein acetyltransferase PatZ from *Escherichia coli* is regulated by autoacetylation-induced oligomerization. J. Biol. Chem..

[71] Trievel R.C., Rojas J.R., Sterner D.E., Venkataramani R.N., Wang L., Zhou J. (1999). Crystal structure and mechanism of histone acetylation of the yeast GCN5 transcriptional coactivator. J. Biol. Chem..

[72] Christensen D.G., Meyer J.G., Baumgartner J.T., D’souza A.K., Payne S.H., Kuhn M.L. (2018). Identification of novel protein lysine acetyltransferases in *Escherichia coli*. mBio.

[73] Shevchenko A., Tomas H., Havliš J., Olsen J.V., Mann M. (2007). In-gel digestion for mass spectrometric characterization of proteins and proteomes. Nat. Protoc..

[74] Sparta K.M., Krug M., Heinemann U., Mueller U., Weiss M.S. (2016). XDSAPP2.0. J. Appl. Crystallogr..

[75] Skubak P., Arac D., Bowler M.W., Correia A.R., Hoelz A., Larsen S. (2018). A new MR-SAD algorithm for the automatic building of protein models from low-resolution X-ray data and a poor starting model. IUCrJ.

[76] Cowtan K. (2006). The Buccaneer software for automated model building. 1. Tracing protein chains. Acta Crystallogr. D Biol. Crystallogr..

[77] McCoy A.J., Grosse-Kunstleve R.W., Adams P.D., Winn M.D., Storoni L.C., Read R.J. (2007). Phaser crystallographic software. J. Appl. Crystallogr..

[78] Emsley P., Lohkamp B., Scott W.G., Cowtan K. (2010). Features and development of Coot. Acta Crystallogr. D Biol. Crystallogr..

[79] Murshudov G.N., Skubák P., Lebedev A.A., Pannu N.S., Steiner R.A., Nicholls R.A. (2011). REFMAC5 for the refinement of macromolecular crystal structures. Acta Crystallogr. D Biol. Crystallogr..

[80] Williams C.J., Headd J.J., Moriarty N.W., Prisant M.G., Videau L.L., Deis L.N. (2018). MolProbity: More and better reference data for improved all-atom structure validation. Protein Sci..

[81] Hunter J.D. (2007). Matplotlib: a 2D graphics environment. Comput. Sci. Eng..

[82] Goddard T.D., Huang C.C., Meng E.C., Pettersen E.F., Couch G.S., Morris J.H. (2018). UCSF ChimeraX: meeting modern challenges in visualization and analysis. Protein Sci..

[83] Krissinel E., Henrick K. (2007). Inference of macromolecular assemblies from crystalline state. J. Mol. Biol..

[84] Miao Y., Feher V.A., McCammon J.A. (2015). Gaussian accelerated molecular dynamics: unconstrained enhanced sampling and free energy calculation. J. Chem. Theory. Comput..

[85] Laemmli U.K. (1970). Cleavage of structural proteins during the assembly of the head of bacteriophage T4. Nature.

[86] Bradford M.M. (1976). A rapid and sensitive method for the quantitation of microgram quantities of protein utilizing the principle of protein-dye binding. Anal. Biochem..

[87] Pettersen E.F., Goddard T.D., Huang C.C., Couch G.S., Greenblatt D.M., Meng E.C. (2004). UCSF Chimera—a visualization system for exploratory research and analysis. J. Comput. Chem..

[88] Jorgensen W.L., Chandrasekhar J., Madura J.D., Impey R.W., Klein M.L. (1983). Comparison of simple potential functions for simulating liquid water. J. Chem. Phys..

[89] Machado M.R., Pantano S. (2020). Split the charge difference in two! a rule of thumb for adding proper amounts of ions in MD simulations. J. Chem. Theory. Comput..

[90] Maier J.A., Martinez C., Kasavajhala K., Wickstrom L., Hauser K.E., Simmerling C. (2015). ff14SB: improving the accuracy of protein side chain and backbone parameters from ff99SB. J. Chem. Theory Comput..

[91] Le Grand S., Götz A.W., Walker R.C. (2013). SPFP: speed without compromise—a mixed precision model for GPU accelerated molecular dynamics simulations. Comput. Phys. Commun..

[92] Ryckaert J.P., Ciccotti G., Berendsen H.J.C. (1977). Numerical integration of the cartesian equations of motion of a system with constraints: molecular dynamics of n-alkanes. J. Comput. Phys..

[93] Essmann U., Perera L., Berkowitz M.L., Darden T., Lee H., Pedersen L.G. (1995). A smooth particle mesh Ewald method. J. Chem. Phys..

[94] Roe D.R., Cheatham T.E. (2013). PTRAJ and CPPTRAJ: software for processing and analysis of molecular dynamics trajectory data. J. Chem. Theory Comput..

