# Supplementary material for: Biochemical and structural characterization of a GNAT superfamily protein acetyltransferase from Helicobacter pylori
Source: J Biol Chem. 2025 Jun 10;301(7):110356. doi: 10.1016/j.jbc.2025.110356 (PMC12273574; doi:10.1016/j.jbc.2025.110356)
Supplement: Supporting information [file mmc2.pdf]

Supporting Information

Biochemical and structural characterization of a GNAT superfamily protein acetyltransferase from *Helicobacter pylori*

Venkatareddy Dadireddy[1 #], Amrendra Kumar[2 #], Sumith Kumar[2], Siddhartha P. Sarma[3], Pranjal Mahanta[4], Suryanarayanarao Ramakumar[1 *], and Rao N. Desirazu[2 *]

[1] Department of Physics, Indian Institute of Science, Bangalore, India,

[2] Department of Biochemistry, Indian Institute of Science, Bangalore, India,

[3] Molecular Biophysics Unit, Indian Institute of Science, Bangalore, India, and

[4] School of Biological Sciences, National Institute of Science Education and Research, Bhubaneswar, India

*To whom correspondence should be addressed dnrao@iisc.ac.in, ramak@alum.iisc.ac.in

[#]These authors contributed equally to this work.

Contents

Supporting results 2

Oligomeric status of HP0935........ 2

HP0935 structure determination 3

ACO possibly facilitates α1-α2 loop transition from distal to proximal conformation 3

Supporting experimental procedures 5

Purification of proteins 5

HP0935 and its variants........ 5

DprA and its variants 7

Analytical gel filtration chromatography........ 7

Calculation of residue-wise RMSD 8
Molecular dynamics simulations 8
Simulation trajectory analysis 9
References 10
Supporting figures 11
Supporting tables 25

Supporting results

Oligomeric status of HP0935

Most GNAT members occur as dimers. However, monomeric, trimeric, tetrameric, and hexameric acetyltransferases have also been reported (45). To determine the correct oligomeric status of HP0935, analytical gel filtration chromatography was performed with tag-removed HP0935 (calculated molecular weight: ~18 kDa). HP0935 eluted as a single, sharp peak corresponding to an apparent molecular weight of 17.35 kDa. Reinjection of the eluted fraction resulted in a similar elution profile. (Fig. S6A), indicating that HP0935 exists as a monomer in solution. We further investigated whether the functional unit of the enzyme is also a monomer. The rate of acetylation using lysine as a substrate was assayed at different concentrations of HP0935. A TLC-based experiment, as described previously, was conducted using increasing concentrations (100 nM - 8 μM) of HP0935 in a reaction containing 20 mM lysine and 50 μM ^{14}C-ACO. Aliquots (5 μL) were withdrawn at regular time intervals and spotted on TLC silica plate to stop the reaction. The acetylated products and unreacted ^{14}C-ACO from the reaction mixture were separated on TLC silica plate and quantified using phosphor imaging followed by densitometry analysis. As seen in Fig. S6B the rate of acetylation versus enzyme concentration showed a linear relationship, indicating that the acetylation reaction is a first order with respect to enzyme concentration. These results confirm that HP0935 functions as a monomeric enzyme.

HP0935 structure determination

HP0935 crystals were grown in two different crystallization conditions (see Experimental procedures, Fig. S9A-C). The initial structure was determined by experimental phasing, and a high-resolution structure was subsequently obtained by molecular replacement (see Experimental procedures). The apo-HP0935 structure was solved at 2.00 Å resolution in the C2 space group, with three molecules (A-C) in the asymmetric unit (ASU). Residues 35-36 and 143-147 (chain A), 34-37 and 143-149 (chain B), and 32-36 and 143-147 (chain C) were not modeled due to the missing electron density. No significant structural changes were observed among the molecules of ASU, as indicated by residue-wise Cα root mean square distance (RMSD) and mean Cα RMSD i.e., 0.45(0.08, SD) Å.

The ACO-HP0935 structure was determined to 2.40 Å resolution in the $P6_1$ space group, with four molecules (A-D) in the ASU. The residue-wise RMSD and mean Cα RMSD value 0.29(0.05) Å indicate that there are no significant structural differences among the molecules of ACO-HP0935 ASU. The electron density was continuous for all polymer chains.

A well-defined density was observed at the putative ACO-binding site in the Fourier difference map (F_o-F_c) contoured at 3.0 σ, for all four molecules in the ASU, and the density was modeled as ACO (Fig. S7B). Structure refinement statistics are presented in Table S2. Crystal packing analysis performed using PISA (83) revealed that the interfaces among the molecules of ASU of apo- and ACO-HP0935 crystal structures are not biologically significant, supporting the conclusion that HP0935 is monomeric in solution, as also demonstrated by analytical gel filtration chromatography (Fig. S6A).

ACO possibly facilitates α1-α2 loop transition from distal to proximal conformation

The effect of ACO binding on the α1-α2 loop transition, distal to proximal, was investigated using Gaussian accelerated molecular dynamics (GaMD) simulations, which enable exploration of conformational states

beyond conventional molecular dynamics (cMD) timescales (84). Two simulation systems were built from the apo-HP0935 crystal structure: system I (apo-HP0935) and system II (apo-HP0935 + ACO) (see Supporting experimental procedures). These simulations, each run for 1 μs, assessed loop dynamics in the absence and presence of ACO. The structural stability of protein during simulations was confirmed via RMSD and radius of gyration (RoG) (Fig. S14).

Loop transition was quantified using the RMSD of the α1-α2 loop relative to its proximal conformation in the ACO-HP0935 structure and the distance between F29 in the trajectory and its proximal position (see Supporting experimental procedures). Lower values indicate a shift towards proximal conformation. In the crystal structures, the distal loop conformation deviates by 4.00(0.35) Å from the proximal conformation. In system I, the α1-α2 loop showed a high RMSD (mean: 9.29(1.34) Å; mode: 8.75 Å), nearly twice that observed in crystal structures (Fig. S12A). In system II, this deviation was significantly reduced (mean: 5.97(1.07) Å; mode: 5.47 Å), indicating ACO's stabilizing effect on the proximal-like conformation (Fig. S12B). Though this value is slightly higher than crystal structures, it is much lower than system I. To assess whether ACO promotes full loop transition, we measured the percentage of trajectory frames where the α1-α2 loop RMSD was ≤2.0 Å from the proximal conformation. In system II, 0.0016% of frames were within this threshold, whereas no such transitions were observed in system I (Fig. S12A-B).

Residue F29 undergoes a 9.44 Å shift during loop transition in the crystal structures. In system I, the average F29 distance from the proximal position was 12.06(3.07) Å (mode: 12.13 Å), exceeding the crystal structure deviation (Fig. S12C). In system II, these values were lower (mean: 8.68(3.26) Å; mode: 6.41 Å), suggesting that in the presence of ACO the F29 distance reduced as compared to system I. Further, 0.035% of trajectory frames in system II had F29 within 2.0 Å of the proximal position, which is about 8-fold higher compared to system I (0.0042%) (Fig. S12C-D). These findings suggest that ACO influences α1-α2 loop dynamics, reducing deviation from the proximal conformation and could induce distal to

proximal transition. The transition likely occurs in solution, while crystal packing further stabilizes the proximal loop, as observed in the ACO-HP0935 structure (Fig. S11).

Supporting experimental procedures

Purification of proteins

HP0935 and its variants

E. coli Rosetta™(DE3) pLysS cells were transformed with pET15b-*hp0935* construct. The transformed cells were plated on LB agar followed by incubation at 37°C overnight. Five colonies from the transformed plate were inoculated in 4 mL of LB broth containing ampicillin (100 µg/mL) and chloramphenicol (34 µg/mL). Two secondary cultures were prepared for each colony by inoculating 1% of the primary culture into 4 mL of LB media containing ampicillin (100 µg/mL) and chloramphenicol (34 µg/mL). Two cultures for each colony were marked as uninduced and induced. The culture was grown till OD_{600} reached 0.6 and then induced with 0.5 mM isopropyl ß-D-1-thiogalactopyranoside (IPTG) and further incubated at 37°C for 4 hr. Cells were harvested by centrifugation at 5,000×g for 5 min and lysed in Buffer A (50 mM Tris-Cl (pH 7.4), 100 mM NaCl, 10 mM EDTA, 10% (v/v) glycerol, and 2 mM β-mercaptoethanol (BME)). The supernatant was separated and collected by centrifuging at 20,000×g for 30 min. An equal volume of supernatant from each colony was loaded on 0.1% SDS-10% PAGE to check the small-scale overexpression of His_6-tag HP0935. The colony showing optimal overexpression was used for large-scale expression under identical conditions. The larger culture was grown in LB medium supplemented with ampicillin (100 µg/mL) and chloramphenicol (34 µg/mL), induced at OD_{600} of 0.6 with 0.5 mM IPTG, and further incubated for 4 hr at 37°C/180 rpm. Cells were harvested at 5,000×g for 10 min. The cell pellet was resuspended and lysed in buffer A containing 2 mM imidazole, 1 mM phenylmethylsulfonyl fluoride (PMSF), and 0.05% triton-X 100. After centrifugation at 20,000×g, 4°C for 30 min, the supernatant was loaded onto a pre-equilibrated prepacked Ni-NTA column (GE Healthcare). The column was washed with

50 mL of Buffer A containing 10 mM imidazole and the protein was eluted with Buffer A containing 10 mM to 300 mM imidazole gradient. Purity was checked on 0.1% SDS- 10% PAGE and the fractions showing protein of interest were pooled and subjected to size-exclusion chromatography using Superdex 200 HiLoad 16/600 column (GE Healthcare), equilibrated in Buffer A with 300 mM NaCl. The purified protein was dialyzed against storage buffer (50 mM Tris-Cl (pH 7.4), 100 mM NaCl, 50% glycerol, and 2 mM BME), at 4°C. Protein purity was assessed by SDS-PAGE and Coomassie brilliant blue staining (85). Protein concentration was determined by Bradford's assay using bovine serum albumin as standard (86). All the purification steps were performed at 4°C.

GST-tagged HP0935 was expressed and purified from *E. coli* Rosetta (DE3) pLysS cells. Cells harboring pGEX_4T2-*hp0935* construct were grown in LB broth media containing ampicillin (100 μg/mL) and chloramphenicol (34 μg/mL) at 37°C with shaking (180 rpm). Cells were induced at OD_{600} between 0.6-0.8 with 0.5 mM IPTG for 4 hr at 37°C. Cells were harvested by centrifugation at 5,000×g for 10 min, re-suspended, and lysed in PBS (140 mM NaCl, 2.7 mM KCl, 10 mM Na_2HPO_4, 1.8 mM KH_2PO_4, and pH 7.3) containing 1 mM PMSF, and 0.05% triton-X 100. The supernatant was collected by centrifugation at 20,000×g, 4°C for 30 min and loaded onto the pre-equilibrated GSTrap HP column (GE Healthcare, USA). The column was washed with 50 mL of PBS, and the protein was eluted with 50 mM Tris-HCl (pH 8.2) containing 10 mM reduced glutathione. Fractions containing the protein were pooled and further purified using Superdex 200 HiLoad 16/600 column (GE Healthcare, USA) column, pre-equilibrated with 50 mM Tris-HCl (pH 7.4), 300 mM NaCl, 10 mM EDTA, 10% (v/v) glycerol, and 2 mM BME. The purified protein was dialyzed against a storage buffer (50 mM Tris-HCl, pH 7.4, 100 mM NaCl, 50% glycerol, and 2 mM BME).

For crystallization, HP0935 protein was expressed from pNIC28-*hp0935* construct in *E. coli* BL21 Star™ (DE3) cells. Transformed cells were grown in 1.6 L of LB broth containing 50 μg/mL of kanamycin at 37°C. Protein expression was induced for 3 hr at 37°C with 0.5 mM of IPTG when OD_{600} reached 0.8. Cells were

harvested by centrifugation at 6,000×g for 10 min at 4°C. The protein was purified using the affinity-based immobilized-metal ion chromatography (IMAC) method. Cells were resuspended in Buffer B (50 mM Tris-HCl (pH 7.4), 200 mM NaCl, 0.01% Triton X100, 2.5 mM BME, and 1 mM PMSF) containing 5 mM imidazole and cells were lysed by sonication. The lysate was clarified by centrifugation at 20,000×g for 30 min and passed through a 0.2 µm syringe filter before loading onto a 1 ml HisTrap™ column (GE Healthcare, USA), pre-equilibrated with Buffer B. The column was washed with Buffer C (50 mM Tris-HCl (pH 7.4), 1 M NaCl, 5 mM BME, and 30 mM imidazole) followed by Buffer B containing 30 mM imidazole. Elution was performed using Buffer B containing 300 mM imidazole. The His_6-tag was cleaved using tobacco etch virus (TEV) protease during dialysis in cleavage buffer (50 mM Tris-HCl (pH 8.0), 100 mM NaCl and 5 mM BME). Tag and TEV protease were removed using HisTrap™ to obtain pure protein. Protein was concentrated and buffer was exchanged to storage buffer (50 mM Tris-HCl (pH 8.0), 150 mM NaCl, and 2.5 mM BME) using a 10 kDa molecular weight cut-off (MWCO) centrifugal filter. The protein was either used immediately for crystallization or flash-frozen in liquid nitrogen and stored at -80° C.

DprA and its variants

DprA and its mutants DprA-K133R and DprA-K133Q were purified as previously described in (43).

Analytical gel filtration chromatography

Gel filtration chromatography was performed using Superdex 200 10/300 column (GE Healthcare) connected with AKTA Basic 10 liquid chromatography system (GE Healthcare) to determine the molecular mass of the HP0935 protein. Chromatography was carried out in 50 mM Tris-Cl (pH 7.4) buffer containing 5 m MBME and 150 mM NaCl at a flow rate of 0.5 mL/min. The column was calibrated using the following standard molecular weight markers (Bio-Rad, USA): Thyroglobulin (600 kDa), γ-globulin (158 kDa), ovalbumin (44 kDa), myoglobin (17 kDa), and vitamin B_{12}(1.35 kDa). The elution volume (V_e) of HP0935 protein and standard molecular weight markers were determined. The void volume (V_o) of the column was determined by using Blue dextran. The elution profile of HP0935 was recorded at 230 nm and 280 nm. A

standard curve was obtained by plotting the logarithm of the molecular mass of standards against their corresponding V_e/V_o values.

Calculation of residue-wise RMSD

For each crystal structure (apo-HP0935 or ACO-HP0935), residue-wise Cα RMSD values between the chains of ASU were calculated using Python script (https://pymolwiki.org/index.php/RmsdByResidue), executable in PyMol. The residue-wise Cα RMSD between chains of apo- and ACO-HP0935 was also calculated using the same approach. Before calculating the RMSD, the chains were aligned using the *align* command. The residue-wise Cα RMSD is defined as the distance between the Cα atoms of aligned chains at the same residue position. The overall Cα RMSD between the chains was derived from the residue-wise values using the following equation.

$$\text{Overall C}\alpha\text{ RMSD} = \sqrt{\frac{\sum_{i=1}^{N} \text{residue-wise RMSD}_i{}^2}{N}}$$

The mean Cα RMSD was also calculated for all chain pairs. RMSD values were computed for residues 2-161.

Molecular dynamics simulations

The crystal structure of apo-HP0935 was used to build the simulation systems. Missing residues were modeled using Coot (78) . Two simulation systems were built, namely, system I – apo-HP0935, and system II – apo-HP0935 with ACO modeled in its binding cleft (apo-HP0935 + ACO). To model ACO binding, apo- and ACO-HP0935 structures were superposed, and the ACO molecule was transferred to apo-HP0935. The resulting structure was energy minimized using Chimera (87). Each structure was placed in a truncated octahedron solvation box with 12.0 Å padding from the solute surface and solvated with the TIP3P water model (88). The ionic strength was set to 0.15 M with Na^+ and Cl^- ions (89). All systems were parameterized using AMBER ff14SB forcefield (90).

Energy minimization was performed with 1,000 steps of steepest descent followed by 9,000 steps of conjugate gradient minimization with 10.0 kcal/mol restraints on the solute atoms. The systems were heated from 0 to 300 K over 100 ps, followed by 1 ns equilibration under the NVT ensemble with 5.0 kcal/mol restraint force. This was followed by 3 ns NPT equilibration with gradual release of restraints from 5.0 to 0.0 kcal/mol. Before GaMD simulations (82), each system was subjected to 50 ns of unrestrained conventional MD (cMD) simulations. Following cMD simulation, to proceed with GaMD simulations, the system's threshold energy (*E*) was set to maximum potential energy (V_{max}). Potential energy statistics (V_{max}, V_{min}, V_{av}, and σ_V) were collected during 10 ns cMD simulations. A dual-boost scheme (total potential and dihedral boost) was applied with acceleration parameters σ_{0P} and σ_{0D} set to 6.0 kcal/mol. Boost parameters were updated every 100 ps during 40 ns of GaMD equilibration. Final GaMD production runs were performed for 1 µs for each system. Simulations were carried out using the GPU-accelerated version of *pmemd.cuda* engine of AMBER20 package (91). Periodic boundary conditions were applied. During simulations, all the bonds involving hydrogen atoms were constrained with the SHAKE algorithm (92). Electrostatics were calculated using the Particle Mesh Ewald (PME) method (93) with a non-bonded cutoff distance of 9.0 Å. Langevin dynamics were used to control the temperature with collision frequency (γ) set to 1 ps^{-1}. The pressure was maintained at 1 atom with the help of a Monte Carlo barostat with a pressure relaxation time of 2 ps. A 2 fs integration time step was used, and coordinates were saved every 2 ps.

Simulation trajectory analysis

All simulation trajectories were analyzed using CPPTRAJ (94). Water and ions were stripped off the trajectories, and the protein molecule was centered in the simulation box. Backbone RMSD, RMSF (for CA, C, N, O, and H atoms), and RoG were calculated. Before RMSF calculation, the trajectories were aligned to the following secondary structure elements of the crystal structure: residues 52-57, 63-70, 75-80, 95-

106, 112-116, 122-128, 134-137, and 154-158). The backbone RMSD of the α1-α2 loop was computed for all trajectories with respect to both apo- and ACO-HP0935 crystal structures.

References

43. Dwivedi, G. R., Sharma, E., and Rao, D. N. (2013) Helicobacter pylori DprA alleviates restriction barrier for incoming DNA. *Nucleic Acids Res*. **41**, 3274–3288

45. Ud-Din, A. I. M. S., Tikhomirova, A., and Roujeinikova, A. (2016) Structure and functional diversity of GCN5-related N-acetyltransferases (GNAT). *Int J Mol Sci*. **17**, 1018

78. Emsley, P., Lohkamp, B., Scott, W. G., and Cowtan, K. (2010) Features and development of Coot. *Acta Crystallogr D Biol Crystallogr*. **66**, 486–501

83. Krissinel, E., and Henrick, K. (2007) Inference of macromolecular assemblies from crystalline state. *J Mol Biol*. **372**, 774–797

84. Miao, Y., Feher, V. A., and McCammon, J. A. (2015) Gaussian accelerated molecular dynamics: unconstrained enhanced sampling and free energy calculation. *J Chem Theory Comput*. **11**, 3584–3595

85. Laemmli, U. K. (1970) Cleavage of structural proteins during the assembly of the head of bacteriophage T4. *Nature*. **227**, 680–685

86. Bradford, M. M. (1976) A rapid and sensitive method for the quantitation of microgram quantities of protein utilizing the principle of protein-dye binding. *Anal Biochem*. **72**, 248–254

87. Pettersen, E. F., Goddard, T. D., Huang, C. C., Couch, G. S., Greenblatt, D. M., Meng, E. C., and Ferrin, T. E. (2004) UCSF Chimera—A visualization system for exploratory research and analysis. *J Comput Chem*. **25**, 1605–1612

88. Jorgensen, W. L., Chandrasekhar, J., Madura, J. D., Impey, R. W., and Klein, M. L. (1983) Comparison of simple potential functions for simulating liquid water. *J Chem Phys*. **79**, 926–935

89. Machado, M. R., and Pantano, S. (2020) Split the charge difference in two! a rule of thumb for adding proper amounts of ions in MD simulations. *J Chem Theory Comput*. **16**, 1367–1372

90. Maier, J. A., Martinez, C., Kasavajhala, K., Wickstrom, L., Hauser, K. E., and Simmerling, C. (2015) ff14SB: improving the accuracy of protein side chain and backbone parameters from ff99SB. *J Chem Theory Comput*. **11**, 3696–3713

91. Le Grand, S., Götz, A. W., and Walker, R. C. (2013) SPFP: Speed without compromise—A mixed precision model for GPU accelerated molecular dynamics simulations. *Comput Phys Commun*. **184**, 374–380

92. Ryckaert, J. P., Ciccotti, G., and Berendsen, H. J. C. (1977) Numerical integration of the cartesian equations of motion of a system with constraints: molecular dynamics of n-alkanes. *J Comput Phys*. **23**, 327–341

93. Essmann, U., Perera, L., Berkowitz, M. L., Darden, T., Lee, H., and Pedersen, L. G. (1995) A smooth particle mesh Ewald method. *J Chem Phys*. **103**, 8577–8593

94. Roe, D. R., and Cheatham, T. E. (2013) PTRAJ and CPPTRAJ: Software for processing and analysis of molecular dynamics trajectory data. *J Chem Theory Comput*. **9**, 3084–3095

Supporting figures

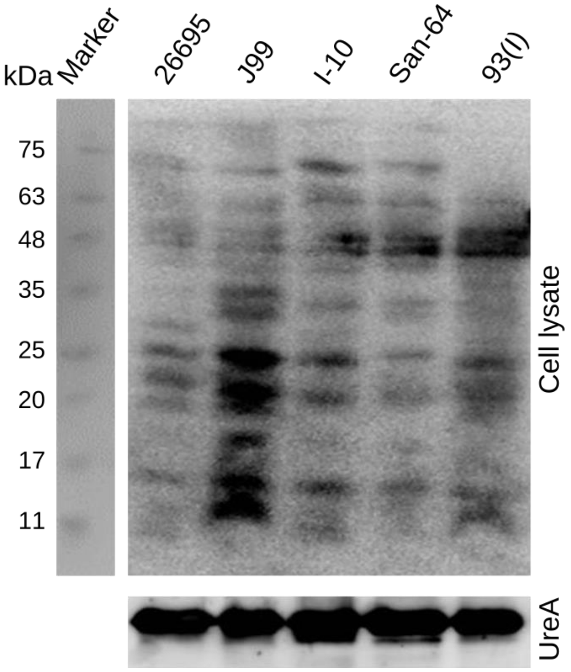

Figure S1: Acetylation in *H. pylori*. Protein acetylation profile in different *H. pylori* strains (26695, J99, I-10, San-64, and 93(I)). *H. pylori* cell lysate was separated on SDS-PAGE and the acetylated proteins were probed by western blot using anti-acetyl lysine antibody. Urease α-subunit (UreA, 26 kDa), a constitutively expressed protein in *H. pylori* was taken as a loading control.

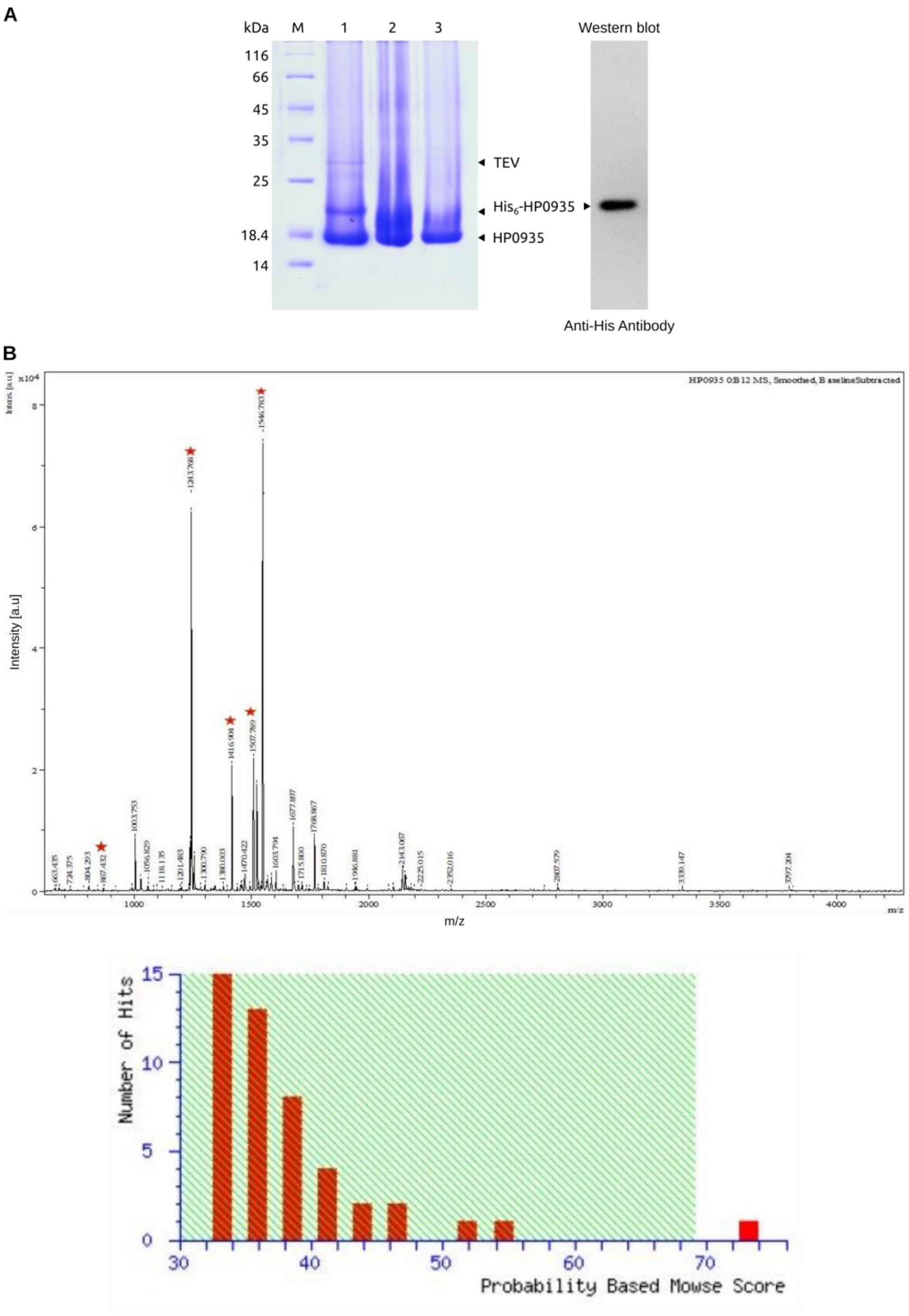

Figure S2: Purification of HP0935. A, HP0955 was purified by Ni-NTA chromatography method. His_6-tag was removed using TEV protease. SDS-PAGE gel showing tag-cleaved HP0935 (left panel). Lanes M: Marker, 1: His_6-HP0935 + TEV protease cleavage reaction, 2 and 3: Flow-through of TEV protease cleavage reaction from Ni-NTA column. His_6-HP0935 was probed by western blot method using anti-his tag antibody (right panel). **B,** Peptide mass finger printing analysis of His_6-HP0935 by mass spectrometry (top panel). Mascot analysis of the mass spectra to confirm the identity of His_6-HP0935. Eight peptides corresponding to HP0935 were identified with a sample score of 73 and an expected value of 0.023.

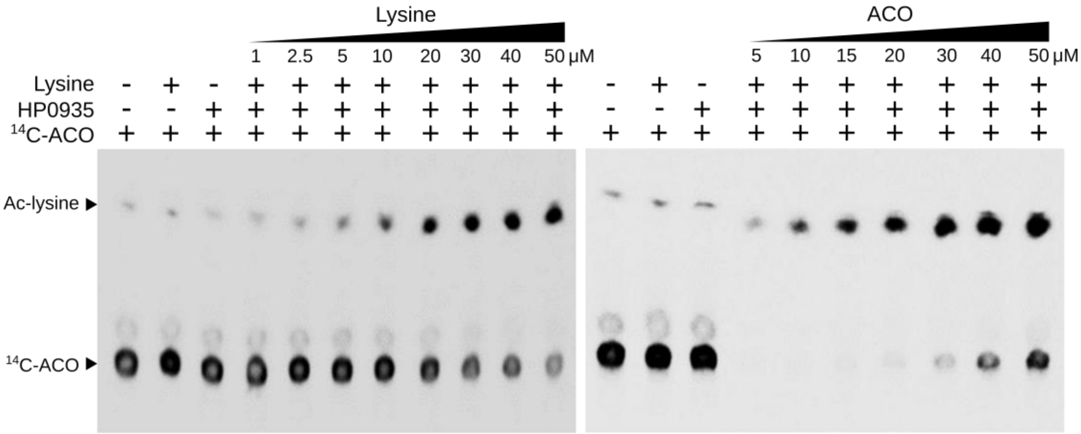

Figure S3: HP0935 enzyme kinetics. Kinetic parameters (K_M and V_{max}) of HP0935 for lysine and ACO were measured. Acetylation reactions containing different concentrations of lysine or ^{14}C-ACO at 800 nM HP0935 concentration were incubated at 30°C for 20 min and the products were separated on silica gel thin layer chromatography (TLC). TLC plates were scored by phosphor imaging.

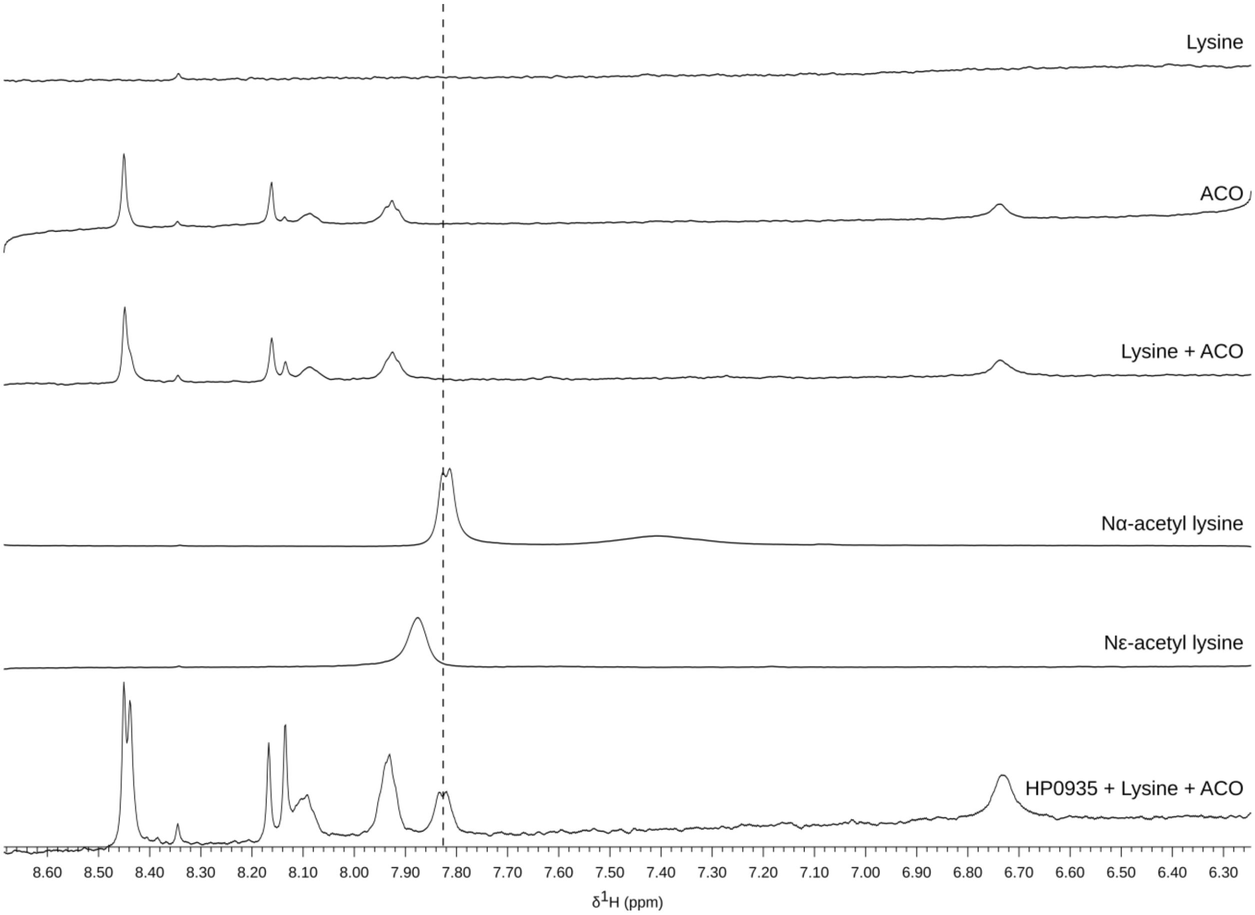

Figure S4: Detection of HP0935 catalyzed lysine acetylation by 1D proton NMR spectroscopy. The broken vertical marks the chemical shift corresponding to Nα acetylation.

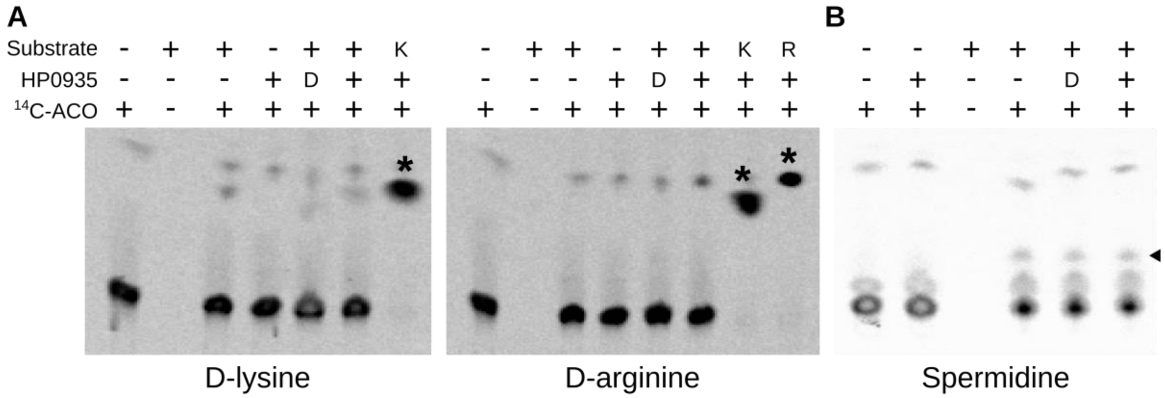

Figure S5: Acetyltransferase activity of HP0935. A, D-lysine and D-arginine. **B,** spermidine. Lysine (K) and arginine (R) were used as positive controls. D: heat-denatured HP0935. Acetylated products are indicated with asterisk (*). Non-enzymatically acetylated spermidine is indicated with an arrowhead.

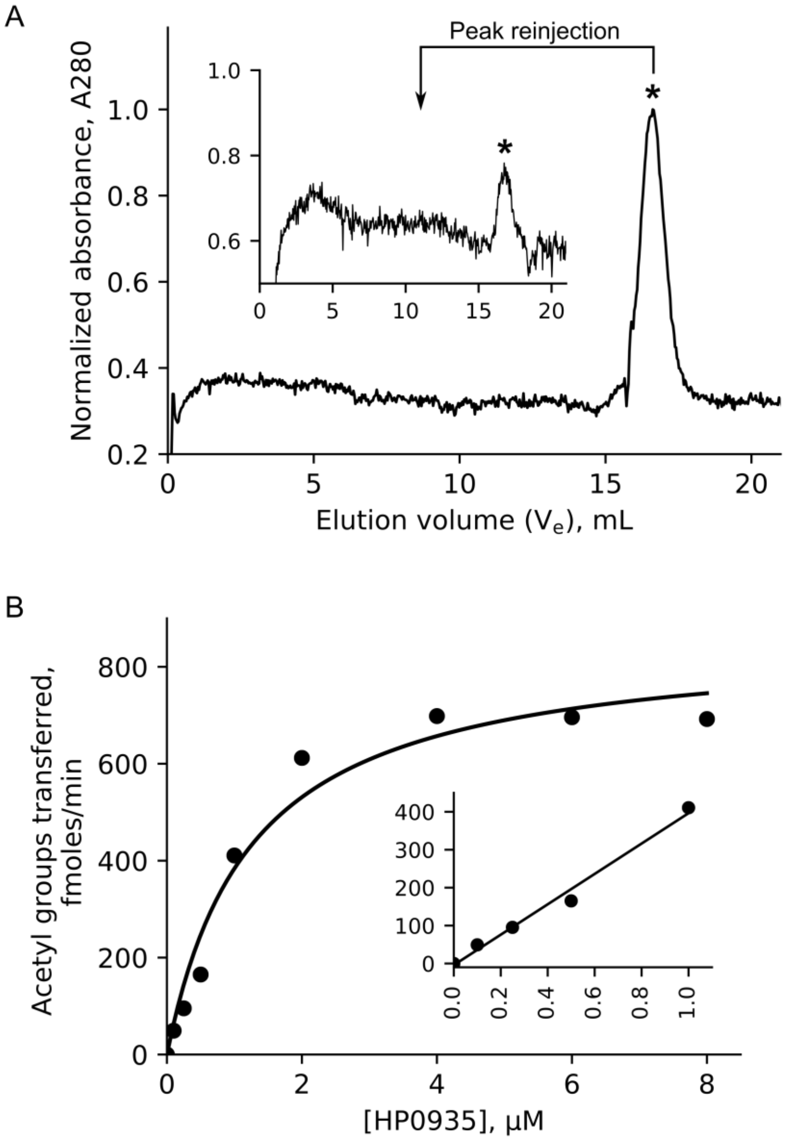

Figure S6: Oligomeric status of HP0935. The oligomeric state of HP0935 was determined by analytical gel filtration chromatography, using 24 mL Superdex 200 Increase 10/300 GL column (Cytiva, USA). **A,** Elution profile of HP0935. HP0935 elutes at 15.3 mL (peak *****), corresponding to its monomeric form (calculated Mr = 18418.33). **B,** Plot showing the effect of HP0935 concentration on the initial velocities. Lysine (20 mM) was incubated with increasing concentration of HP0935 (100 nM 250 nM, 500 nM, 1 μM, 2 μM, 4 μM, 6 μM and 8 μM) in presence of 50 μM ^{14}C-ACO. Inset: initial velocity versus lower concentrations of HP0935 (100 nM, 250 nM, 500 nM and 1 μM) shows the linear progression.

A

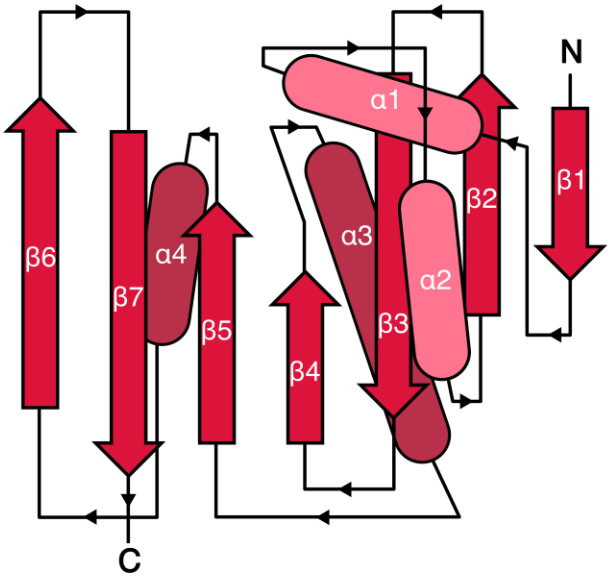

B

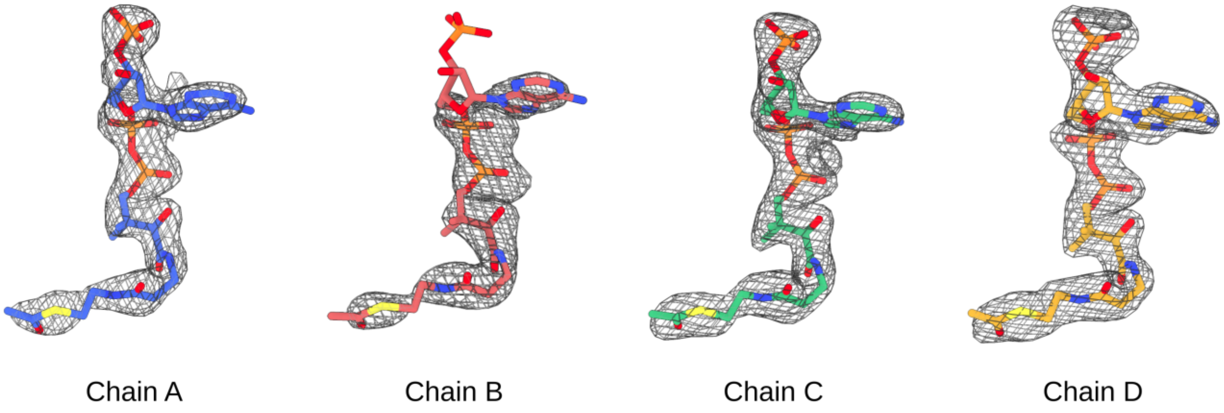

Figure S7: A, The topology diagram of HP0935 structure. **B,** Fourier difference map (F_o-F_c) at 3.0 σ showing electron density at the putative acetyl-coenzyme A (ACO) binding site, modeled as ACO, for all four chains in the asymmetric unit.

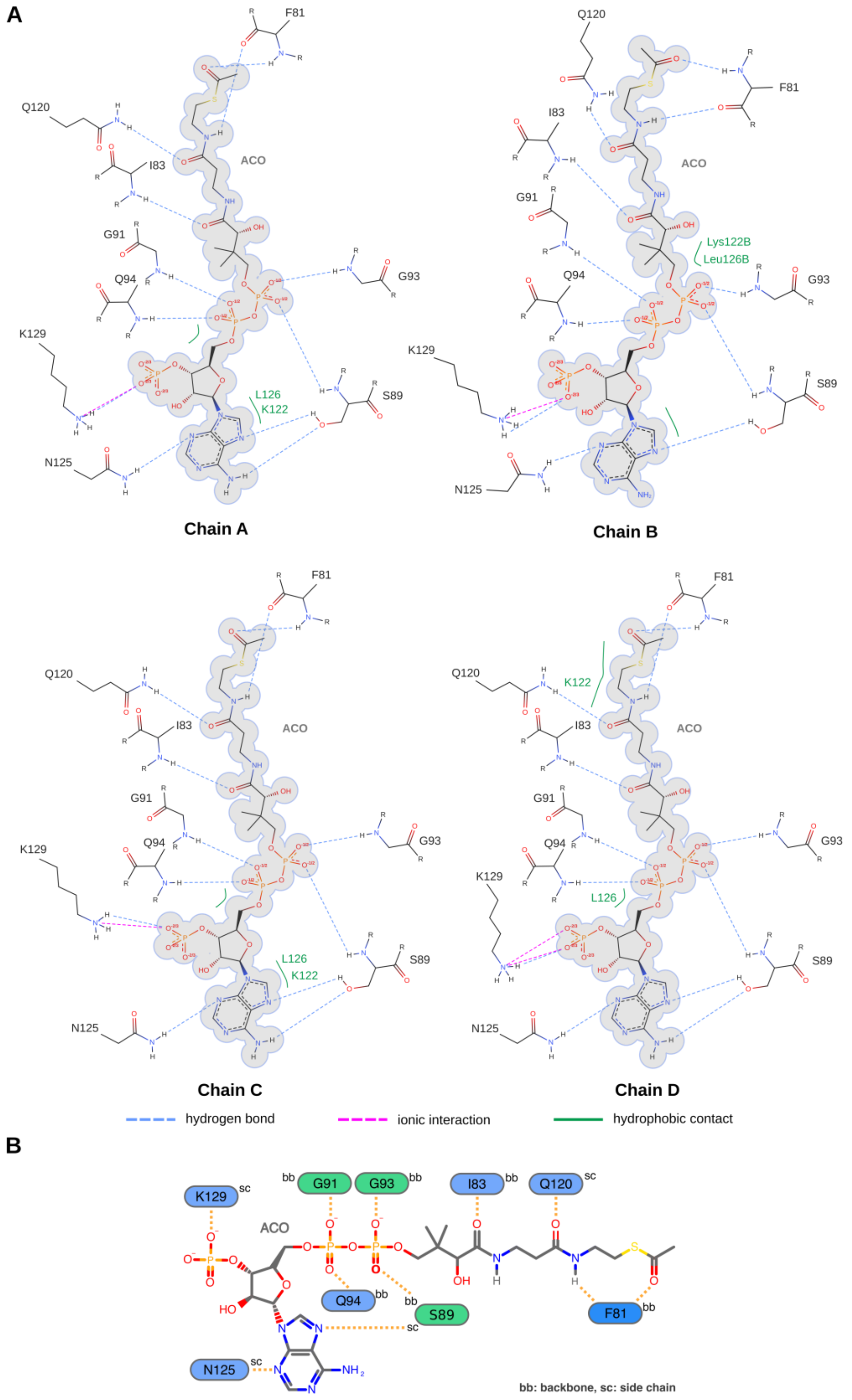

Figure S8: A, Interactions between ACO and HP0935 presented for all four chains of the ASU from ACO-HP0935 crystal structure. **B,** Consensus interactions between HP0935 and ACO from all the chains of the

ASU are shown. Residues from P-loop and the β-bulge are shown in pale green and dodger blue colors, respectively. The symbols bb and sc denote the backbone (bb) and side chain (sc) atoms of an amino acid residue involved in hydrogen bonding with ACO.

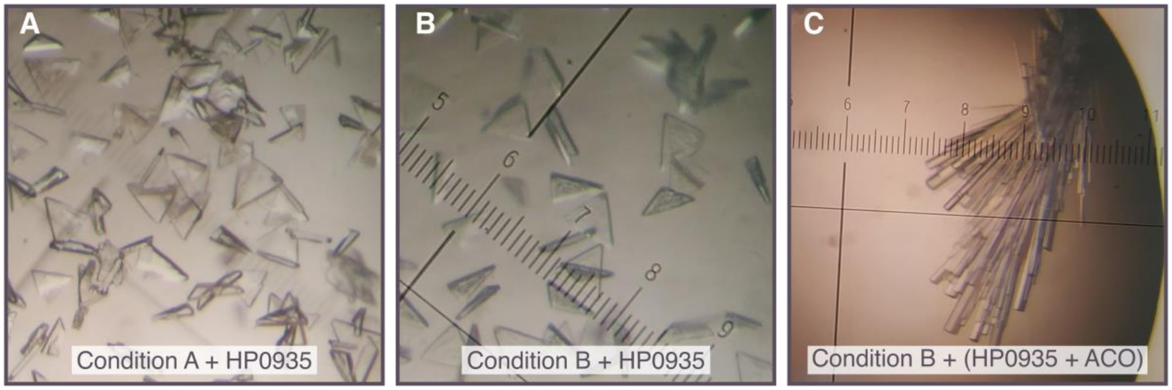

Figure S9: HP0935 crystal morphology in the absence and presence of ACO. **A** and **B,** Apo-HP0935 crystals (triangular plate shaped) obtained from condition A and B. **C,** ACO-HP0935 crystals (needle shaped) from condition B. Condition A: 0.2 M potassium thiocyanate, 0.1 M Bis-Tris propane pH 6.5 and 20% (w/v) polyethylene glycol 3500, and Condition B: 0.02 M magnesium chloride hexahydrate, 0.1 M HEPES pH 7.5 and 22% (w/v) poly(acrylic acid sodium salt) 5,100.

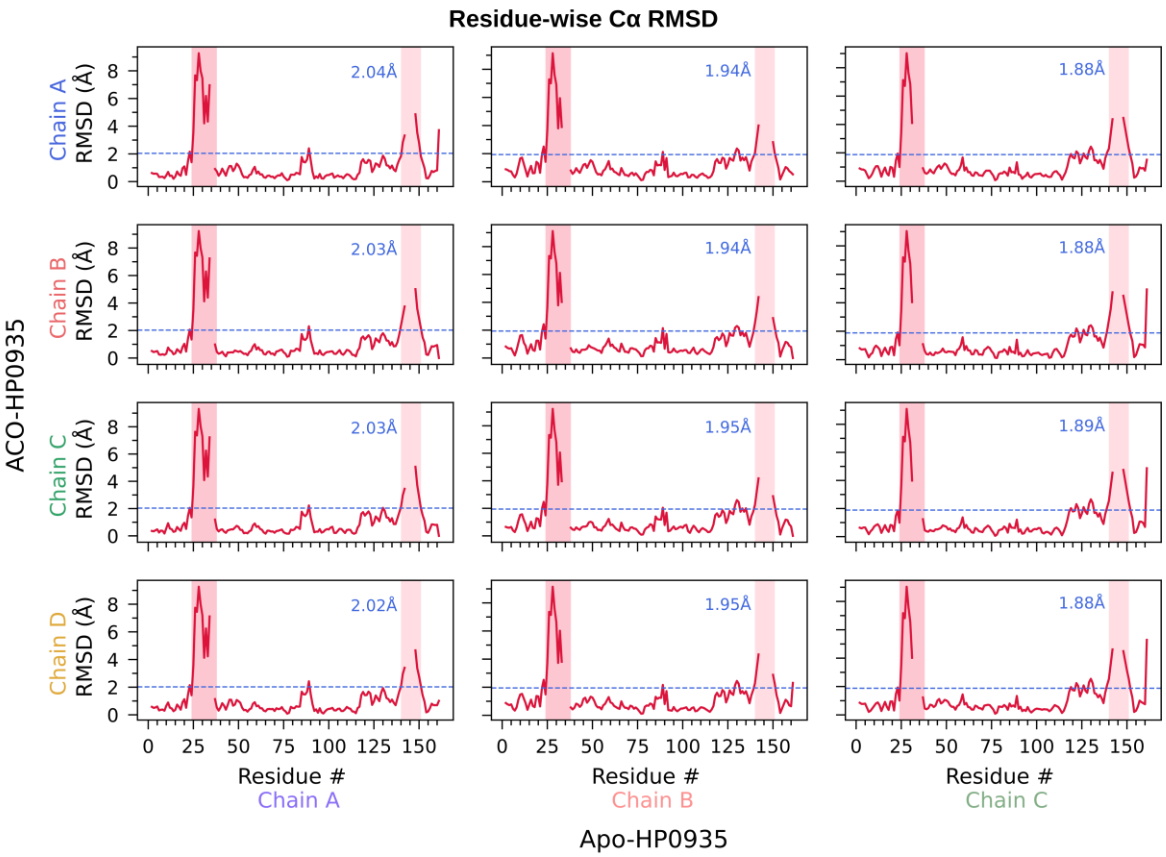

Figure S10: The residue-wise Cα RMSD between the chains of ASUs of apo - and ACO-HP0935 crystal structures. The dark and pale pink shaded areas correspond to α1-α2 and β6-β7 loops, respectively. These regions show relatively high RMSD values. The gaps in the RMSD subplots indicate the missing residues in the apo-HP0935 structure where residue-wise Cα RMSD cannot be computed. The broken horizontal line indicates the overall Cα RMSD between pairs of chains and the corresponding value is shown on the top-right for each subplot. The average Cα RMSD between the chains of apo- and ACO-HP0935 structures is 1.95(0.06) Å (mean(SD)).

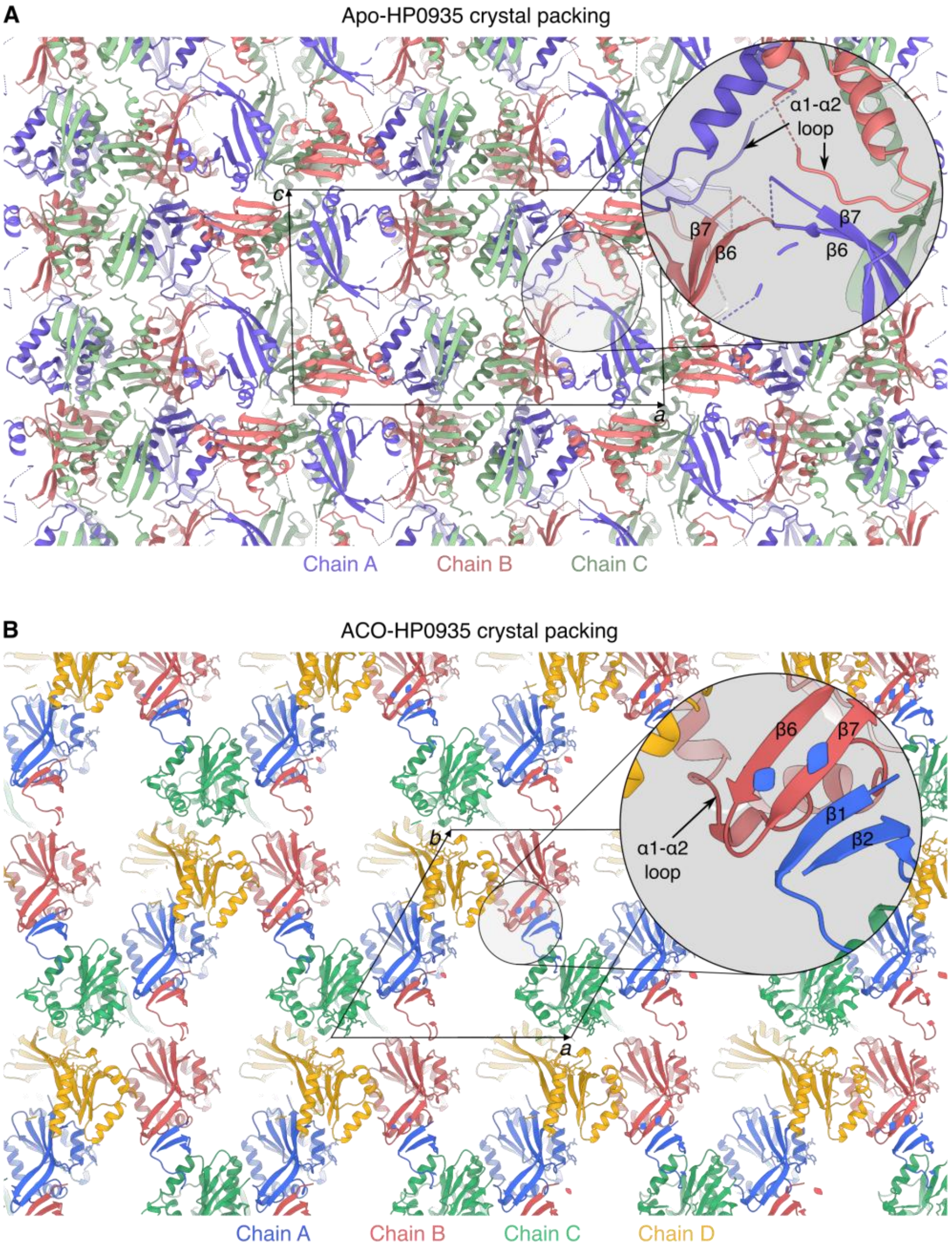

Figure S11: HP0935 crystal packing. A, Apo-HP0935. **B,** ACO-HP0935. Apo- and ACO-HP0935 crystals belong to monoclinic (C2) and hexagonal ($P6_1$) crystal systems, respectively. The α1-α2 and β6 to β7 transition region are solvents exposed in apo-HP0935 crystal form. The β6-β7 hairpin is involved in crystal packing in ACO-HP0935 crystal form. The missing residues are depicted as broken lines.

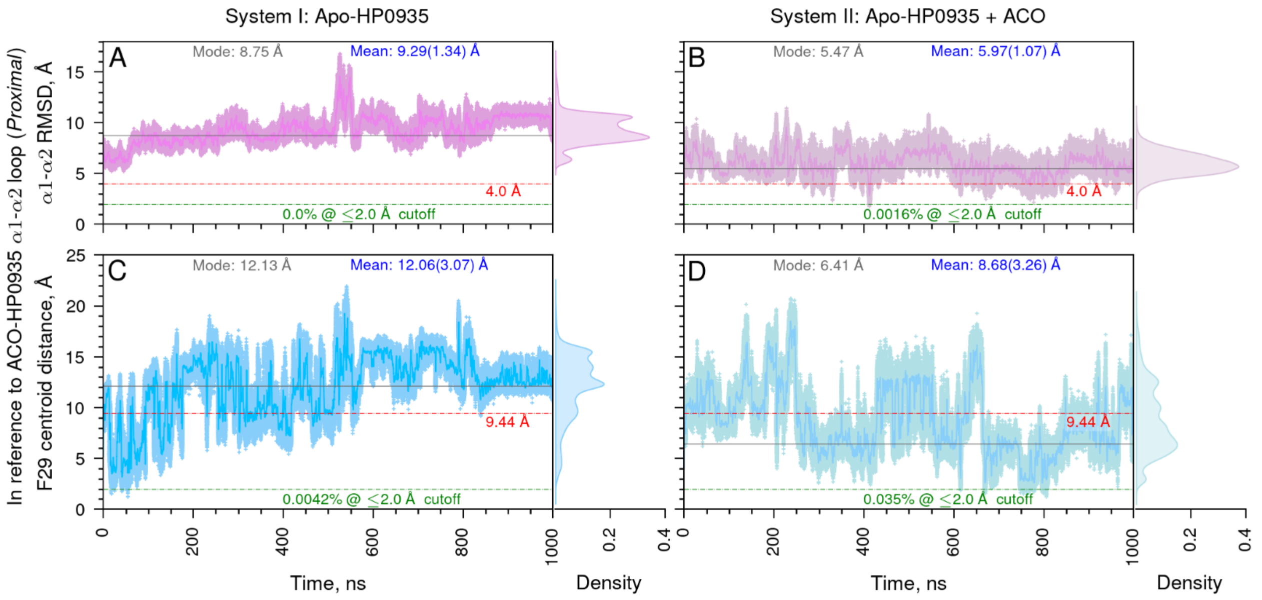

Figure S12: Effect of ACO on transition of α1-α2 loop from distal to proximal conformation. The effect of ACO binding on the distal conformation was tested using GaMD simulations. **A** and **B,** The α1-α2 loop RMSD between the simulation trajectory and the proximal α1-α2 loop conformation (ACO-HP0935 crystal structure) measured for system I (apo-HP0935) and II (apo-HP0935 + ACO). **C** and **D,** Distance deviation of F29 (side chain centroid) position in the simulation trajectory from that of F29 distal position (ACO-HP0935 crystal structure) measured for system I and system II. The mean(SD) and mode (horizontal gray line) values are mentioned for each subplot. The red broken line represents the α1-α2 loop RMSD between distal and proximal conformations in plot **A** and **B**, and distance between F29 positions in distal and proximal conformations in plot **C** and **D**.

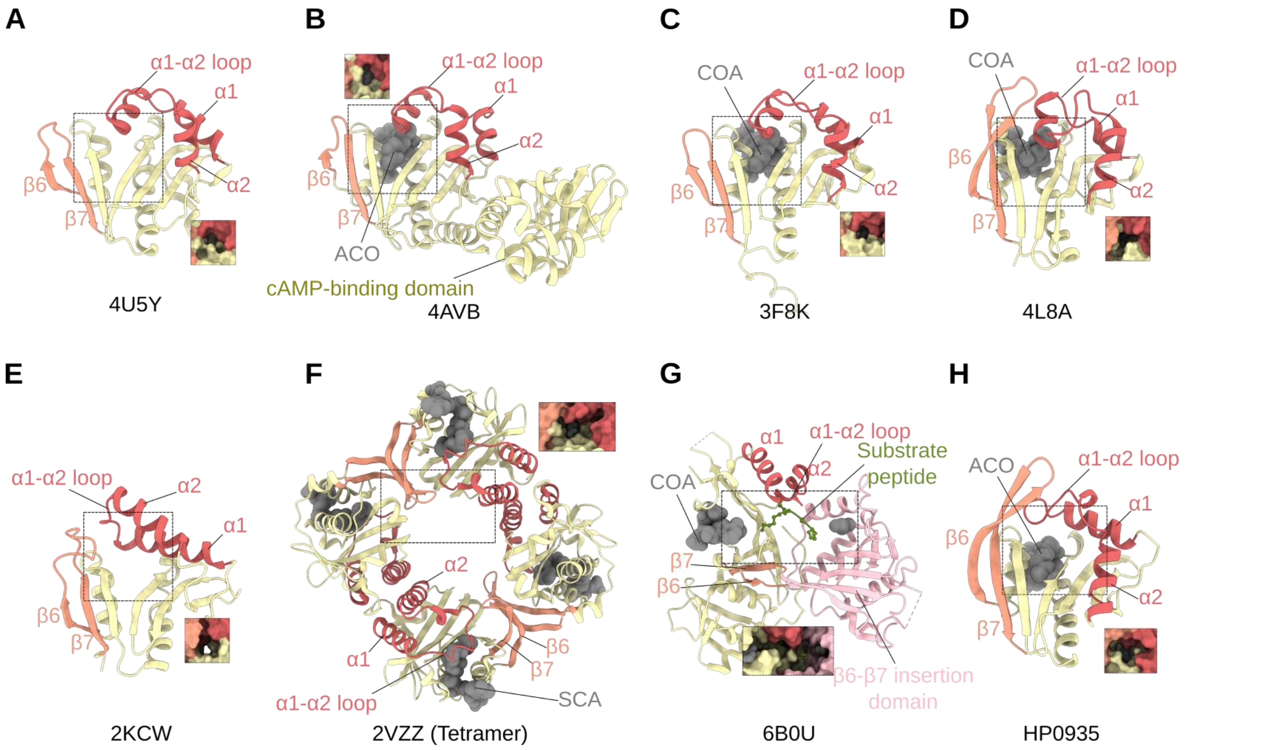

Figure S13: Substrate-binding region in prokaryotic protein acetyltransferases (PATs). The substrate-binding region is mainly formed by α1 and α2 helices (Indian red), α1-α2 loop (Indian red), and β6-β7 region (pale salmon). These structures determine the size and shape of the substrate entry site (the dashed box and its corresponding region is shown in the inset). **A** and **B,** Acetyl coenzyme A synthetase (ACS) acetyltransferase form *Streptomyces lividans* (PDB:4U5Y) and *Mycobacterium tuberculosis* (PDB: 4AVB), respectively. **C,** ALBA acetyltransferase from *Saccharolobus solfataricus* (PDB: 3F8K). **D,** C-terminal lysine acetyltransferase from *Pseudomonas aeruginosa* (PDB: 4L8A). **E,** YjaB from *E. coli* (PDB: 2KCW). **F,** Acetyl/succinyl transferase from *M. tuberculosis* (PDB: 2VZZ). **G,** Eis from *M. tuberculosis* (PDB: 6B0U). **H,** HP0935 (current study). ACO: acetyl coenzyme A, COA: coenzyme A, and SCA: succinyl coenzyme A.

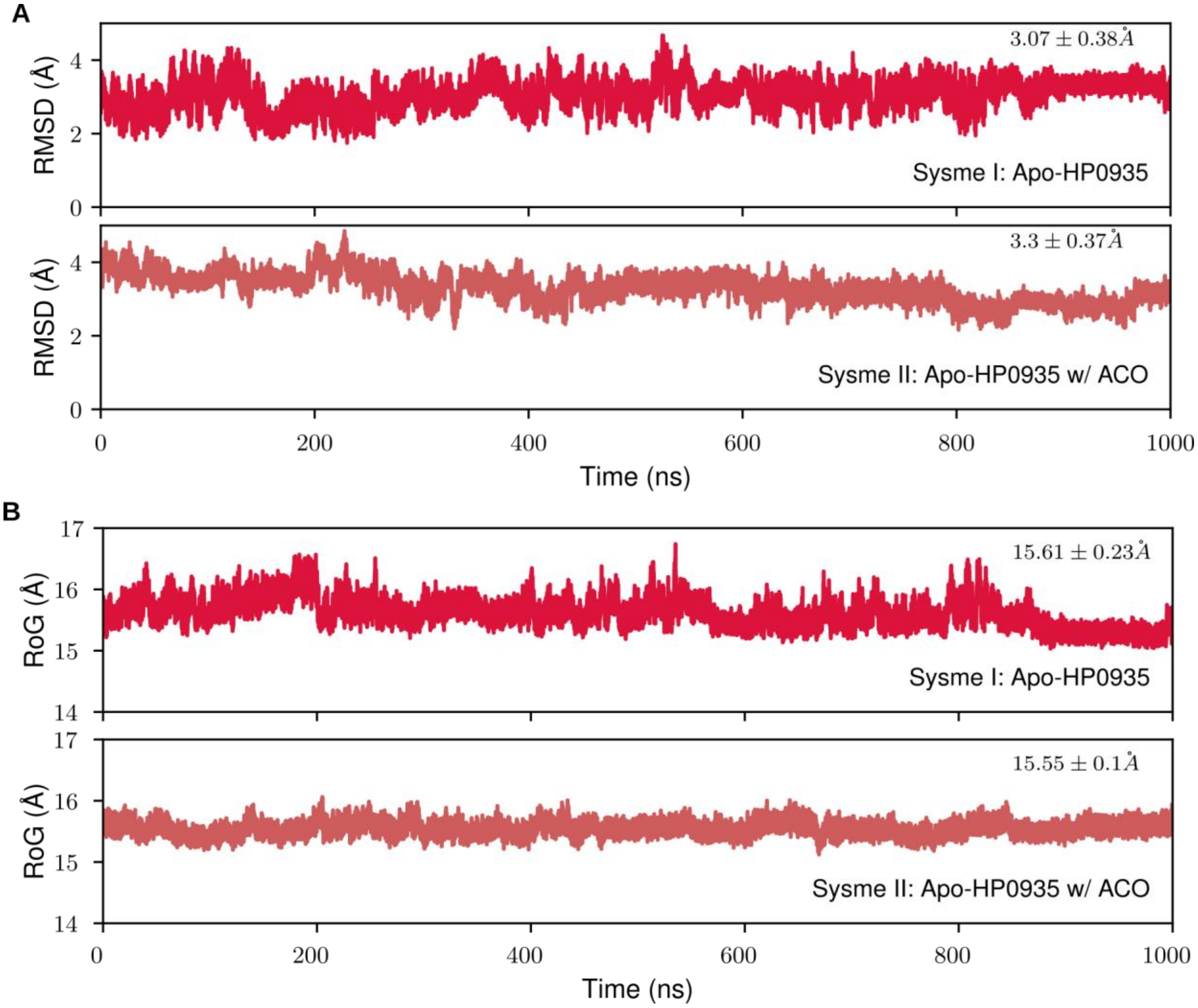

Figure S14: A, Backbone root mean squared distance (RMSD) and **B,** Radius of gyration (RoG) of HP0935 simulation systems I (apo-HP0935) and II (apo-HP0935 + ACO). The backbone RMSD values were calculated in reference to the apo-HP0935 crystal structure.

Supporting tables

Table S1: Identification of GNAT member in *H. pylori* using PSI-BLAST. GNAT protein acetyltransferases (PATs) from different organisms are used in PSI-BLAST.

GNAT PATs	Organism	Sequence identity with HP0935 (%)	Sequence coverage (%)	e-value
MSMEG_5458	*Mycobacterium smegmatis*	27	42	$2e^{-29}$
Rv0998	*M. tuberculosis*	22	42	$1e^{-32}$
YfiQ	*Salmonella enterica*	20	70	$2e^{-32}$
Gcn5	*Saccharomyces cerevisiae*	22	57	$2e^{-28}$

Table S2: X-ray diffraction data collection and refinement statistics.

	**Apo-HP0935 (Iodine-SAD)**	**Apo-HP0935 (Native; PDB: 8IYM)**	**ACO-HP0935 (Native; PDB: 8IYO)**
**Data collection**			
X-ray source	Home source (Cu-Kα)	Home source (Cu-Kα)	ID29, ESRF
Space group	C2	C2	$P6_1$
Cell dimensions			
a, b, c (Å)	117.26, 65.95, 70.94	117.86, 66.17, 70.90	87.80, 87.80, 216.36
α, β, γ (◦)	90, 91.43, 90	90, 91.42, 90	90, 90, 120
Wavelength (Å)	1.542	1.542	1.072
Resolution (Å)	70.92 - 2.42 (2.51-2.42)	70.88 - 2.00 (2.05 - 2.00)	44.08 - 2.40 (2.49 - 2.40)
R_{sym} or R_{merge}	0.137 (0.840)	0.120 (0.586)	0.150 (1.418)
I / σ(I)	16.50 (1.90)	10.4 (2.5)	15.2 (2.7)
Completeness (%)	99.05 (91.75)	99.8 (100.0)	100.0 (99.9)
Redundancy	19.5 (4.5)	7.4 (5.2)	18.3 (19.0)
Wilson B-factor (Å^2)	24.22	16.82	46.76
CC(½)	0.997 (0.562)	0.996 (0.792)	1.000 (0.901)
**Refinement**			
Resolution (Å)		70.88 - 2.00 (2.05 - 2.00)	44.08 - 2.40 (2.49 - 2.40)
No. Reflections all/free		36942/1880	36770/1892
R_{work}/R_{free}		0.216/0.248	0.169/0.202
No. atoms		3949	5434
Protein		3631	5152
Ligand/ion		42	204
Water		276	78
B-factors (Å^2)		24.58	59.63
Protein		24.19	59.75
Ligand/ion		31.96	59.62
Water		28.57	51.18
RMS deviation			
Bond lengths (Å)		0.018	0.015
Bond angle (◦)		2.03	2.19
Ramachandran plot (%)			
Favored		99.32	99.69
Allowed		0.68	0.31
Outlier		0.00	0.00
Rotamer outliers (%)		1.55	3.41
Clash score		5.51	1.41

Values in the parenthesis are for high-resolution shell.

Table S3: Conformational transition of α1- α2 loop in GNAT members.

**UniProt**	**Organism**	**Protein name (Function)**	**PDB ID**	**α1- α2 loop (crystal contacts)**
**Q8GLI5**	*Salmonella typhimurium*	Aminoglycoside 6-N-acetyltransferase typ-Ib11 (antibiotic resistance)	2PR8 (apo)<br>SG: $C222_1$	Long loop;<br>Ordered;<br>Distal;<br>(yes)
			2PRB (COA-bound)<br>SG: $P4_32_12$	Partly disordered;<br>Proximal;<br>(no)
			2QIR (COA and kanamycin)<br>SG: $P4_32_12$	Partly disordered;<br>Proximal;<br>(no)
**V5RDR9**	*Acinetobacter nosocomialis*	FdhC<br>(adds 3-hydroxylbutanoyl group to dTDP-3-amino-3,6-dideoxy-D-galactose)	5KTA (apo)<br>SG: $P6_322$	Ordered;<br>Distal;<br>(yes)
			5KTC (COA and dTDP-3-amino-3,6-dideoxy-D-glucose bound)<br>SG: $P4_1\,2_1\,2$	Moves towards substrate;<br>Proximal;<br>(partly, negligible)
**P9WQG7**	*M. tuberculosis*	Succinyl-CoA transferase; Rv0802c<br>(Antibiotic resistance; protein lysine succinylation)	2VZY (apo)<br>SG: $P2_12_12_1$	Disordered;<br>(no)
			2VZZ (succinly-coA)<br>SG: I222	Moved towards succinyl-coA;<br>Proximal;<br>(no)

Distal: loop is away from ACO/COA/substrate binding cleft.
Proximal: loop moves towards ACO/COA/substrate binding cleft.
SG: Space group

Table S4: List of oligonucleotides used in the current study.

Oligo name	Sequence
HP0935-His-FP	CGCCATATGATGACCATCAAAGTTTTTTCG
HP0935-His-RP	CGGGATCCTCAAGACAAAATCTTTTCC
HP0935-GST-FP	CGGGATCCATGACCATCAAAGTTTTTTCG
HP0935-GST-RP	CCGCTCGAGTCAAGACAAAATCTTTTCC
HP0935-E77Q-FP	CAATCAAACAGAAGCCCAGCTGTGCAAATTCCAC
HP0935-E77Q-RP	GTGGAATTTGCACAGCTGGGCTTCTGTTTGATTG
HP0935-H115A-FP	CTAAAATCTCTCTGGCTGTGAGCAAAAGCC
HP0935-H115A-RP	GGCTTTTGCTCACAGCCAGAGAGATTTTAG
HP0935-Y127F-FP	CAAGGCATGCAACCTCTTTCAAAAGCTGGGTTTTG
HP0935-Y127F-RP	CAAAACCCAGCTTTTGAAAGAGGTTGCATGCCTTG
DprA-K133R-FP	GATTTCATGCCCATTAGAGGCTCTTTTTTAGCC
DprA-K133R-RP	GGCTAAAAAAGAGCCTCTAATGGGCATGAAATC
DprA-K133Q-FP	GATTTCATGCCCATTCAAGGCTCTTTTTTAGCC
DprA-K133Q-RP	GGCTAAAAAAGAGCCTTGAATGGGCATGAAATC
53Forward	CTTGAAGAGGATCCTCGAATTCTCGAGCGCGGTACCCTGCAGTCTTCTGCAAG
